# Large Multicohort Study Reveals a Prostate Cancer Susceptibility Allele at 5p15 Regulating *TERT via* Androgen Signaling-Orchestrated Chromatin Binding of E2F1 and MYC

**DOI:** 10.3389/fonc.2021.754206

**Published:** 2021-11-10

**Authors:** Xiaoming Dong, Qin Zhang, Jinglan Hao, Qianwen Xie, Binbing Xu, Peng Zhang, Haicheng Lu, Qilai Huang, Tielin Yang, Gong-Hong Wei, Rong Na, Ping Gao

**Affiliations:** ^1^ Department of Biochemistry and Molecular Biology, College of Life Sciences, Shaanxi Normal University, Xi’an, China; ^2^ Biocenter Oulu, Faculty of Biochemistry and Molecular Medicine, University of Oulu, Oulu, Finland; ^3^ Fudan University Shanghai Cancer Center, Department of Biochemistry and Molecular Biology, School of Basic Medical Sciences, Shanghai Medical College of Fudan University, Shanghai, China; ^4^ Shandong Provincial Key Laboratory of Animal Cell and Developmental Biology, Department of Animal Science, School of Life Sciences, Shandong University, Qingdao, China; ^5^ Key Laboratory of Biomedical Information Engineering of Ministry of Education, and Institute of Molecular Genetics, School of Life Science and Technology, Xi’an Jiaotong University, Xi’an, China; ^6^ Department of Urology, Ruijin Hospital, Shanghai Jiao Tong University School of Medicine, Shanghai, China

**Keywords:** TERT, rs2853669, prostate cancer, E2F1, MYC, AR signaling, EMT, crpc

## Abstract

Aberrant telomerase reverse transcriptase (*TERT*) expression is crucial for tumor survival and cancer cells escaping apoptosis. Multiple TERT-locus variants at 5p15 have been discovered in association with cancer risk, yet the underlying mechanisms and clinical impacts remain unclear. Here, our association studies showed that the *TERT* promoter variant rs2853669 confers a risk of prostate cancer (PCa) in different ethnic groups. Further functional investigation revealed that the allele-specific binding of MYC and E2F1 at *TERT* promoter variant rs2853669 associates with elevated level of *TERT* in PCa. Mechanistically, androgen stimulations promoted the binding of MYC to allele T of rs2853669, thereby activating *TERT*, whereas hormone deprivations enhanced E2F1 binding at allele C of rs2853669, thus upregulating *TERT* expression. Notably, E2F1 could cooperate with AR signaling to regulate *MYC* expression. Clinical data demonstrated synergistic effects of *MYC*/*E2F1/TERT* expression or with the TT and CC genotype of rs2853669 on PCa prognosis and severity. Strikingly, single-nucleotide editing assays showed that the CC genotype of rs2853669 obviously promotes epithelial–mesenchymal transition (EMT) and the development of castration-resistant PCa (CRPC), confirmed by unbiased global transcriptome profiling. Our findings thus provided compelling evidence for understanding the roles of noncoding variations coordinated with androgen signaling and oncogenic transcription factors in mis-regulating *TERT* expression and driving PCa.

## Introduction

Prostate cancer (PCa) is one of the leading causes of cancer deaths and a highly heritable cancer in men (1). Genetic heritability was estimated to account for 57% of familial risk in PCa (1). Thus, identification of genetic loci in association with PCa risk and pathogenesis, and illustration of the underlying mechanisms are expected to have substantial influence on our understanding of PCa and on the prevention and treatment of the disease. Thanks mainly to genome-wide association studies (GWAS), a substantial amount of single-nucleotide polymorphism (SNP) loci have thus far been identified to be significantly associated with PCa (2). To date, even though the detailed functions of several PCa risk SNPs have been unveiled, the regulatory mechanisms underlying many SNPs remain unidentified. Focusing our research scope on PCa, important oncogene-associated risk SNPs might be a way to speed up our understanding of the functions and the risk of SNPs.

The human telomeres are a safeguard of chromosome ends, whose main function is the maintenance of telomeric DNA length and chromosomal stability (3, 4). Most malignant cells in cancer, including PCa, achieve unlimited replicative capacity, a hallmark of cancer (5), through activating telomerase for telomere maintenance (6, 7). The key rate-limiting element for telomerase activity was found to be *TERT*, encoding an essential catalytic subunit of telomerase (8–10) that is aberrantly expressed in many types of cancer. Understanding the mechanisms of aberrant *TERT* expression is a fundamental question for human cancer, and TERT is also a potential clinical target for improving cancer diagnosis and prognosis (11–13). At present, a variety of transcription factors have been found to regulate the transcription of *TERT* by directly acting on the promoter region of *TERT* or indirectly in the form of complexes. There are two canonical E-box consensus sites at the hTERT core promoter of MYC family transcription factors, which are located at upstream 165 bp and downstream 45 bp relative to the hTERT transcriptional start site (TSS). To date, the mechanisms underlying high *TERT* expression in PCa are still not fully investigated.

In recent years, large-scale genome-wide association studies (GWAS) have made important contributions to the identification of common mutations at *TERT* sites. Many studies have confirmed that somatic mutations and functional SNPs of *TERT* genes are associated with multiple cancer risks. For example, rs2736100 was found to be associated with high risk of glioma, lung adenocarcinoma, and testicular germ cell cancer; rs402710 was found to be associated with lung cancer; and T allele of rs10069690 increased the risk of estrogen-receptor-negative breast cancer. rs2242652, rs7725218, rs2853677, and rs401681 have been reported to be associated with PCa risk. These four SNPs are located at the intron or 3’UTR region of *TERT*. To further validate whether there are SNPs located at the promoter region of *TERT* associated with PCa risk, we focused our research on the SNPs located around *TERT*.

## Materials and Methods

### Study Population, SNP Genotyping, and Quality Control

A total of 209,741 male participants with Caucasian ancestry from UK Biobank (release V3) with GWAS genotyping array data and imputation information were included in the present study. A detailed description of the population of UK Biobank was reported elsewhere (14). Briefly, this is a prospective cohort with genetic and phenotypic data from 500,000 individuals across UK between 40 and 69 years at the age of recruitment. A PCa diagnosis was identified as having a record of PCa from national cancer registries and self-report based on ICD-10 code (C61) before or after recruitment in patients with European Ancestry. The current study was based on the update of the database in December 2020. Genome-wide genotype data with imputation and quality control (QC) were provided by UK Biobank. Genotype information of rs2853669 and its nearby region (± 50 kb) was then obtained from the dataset for further analysis.

An additional independent population with Chinese Han ancestries was also included in the present study for further validation. It included 2,510 male patients from a biopsy cohort based on Chinese Han population (1,100 PCa patients *vs.* 1,410 healthy men as controls). Clinical information was collected including diagnosis of PCa, Gleason Score, and prostate-specific antigen. SNPs were genotyped using the Illumina Asian Screening Array (ASA) for Chinese patients. Subjects were removed from subsequent analyses if they met any of the following criteria: (1) overall genotype call rate <90%; (2) duplicates or familial relationship (PI_HAT > 0.025). Genotypic QC of rs2853669 was also performed (1) if the genotype call rate >95% in the three populations; and (2) if it passes a Hardy–Weinberg Equilibrium test (*p* > 1 × 10^−3^). Finally, this SNP could be further evaluated in these populations.

### Association Study and Fine Mapping

Logistic regression models including additive model, dominant model, and recessive model were used to evaluate the association between the SNPs and different phenotypes of PCa performing by using Plink 1.09. Cox regression survival analyses were performed using R (3.6.3). A two-tailed *p* < 0.05 was considered statistically significant. Fine mapping for the ±50-kb region of rs2853669 was performed based on the association results from UK Biobank using LocusZoom (http://locuszoom.sph.umich.edu/). The linkage disequilibrium (LD) in this region was evaluated by LDlink (15).

### Cell Culture

All cell lines were originally purchased from the American Type Culture Collection (ATCC), and none of these cell lines were found to be contaminated with mycoplasma during our study. HEK293T cells were grown in DMEM (Invitrogen, catalog no. 10569010); LNCaP and 22Rv1 cells were grown in RPMI1640 (Merck, catalog no. R8758). DMEM and RPMI1640 were supplemented with 10% fetal bovine serum (Gibco, catalog no. 10099-141C) and antibiotics (Thermo Fisher Scientific, catalog no. 15140122). RWPE1 cells were grown in Keratinocyte-Serum Free Medium (10724-011, Gibco) with 0.05 µg/ml epidermal growth factor (PHG0311, Gibco), 0.05 mg/ml bovine pituitary extract (02-104, Merck), and antibiotics. In order to study AR activity, we cultured the LNCaP and 22Rv1 cells in charcoal (Merck, catalog no. C6241-5G) stripping media up to at least 48 h. AR activity was induced by treating cells with 100 nM dihydrotestosterone (DHT; dissolved in methanol, Merck, catalog no. D-077-1ML).

### Transfection and Luciferase Promoter Reporter Assays

DNA fragments surrounding rs2853669 (T allele or C allele) were inserted into the pGL3 Basic vector (Promega, catalog no. E1751) (**Supplementary Table 1**). For plasmid transfection in the white 96-well tissue culture plates, 0.4 × 10^5^ 22Rv1 and 0.4 × 10^5^ LNCaP cells per well were applied to reverse transfection with luciferase reporter plasmids together with pGL4.75 (Promega, catalog no. E6931) using X-tremeGENE HP DNA Transfection Reagent (Roche, catalog no. 11668019) following the manufacturer’s instructions. After 48 h, cells were analyzed for luciferase activity using the Dual-Glo^®^ Luciferase Assay System (Promega, catalog no. 06366236001) following the manufacturer’s instructions. All data came from at least three replicate wells, and statistical analyses were performed by a two-tailed *t* test.

### Immunofluorescence

For immunofluorescence analysis, cells were fixed in 4% paraformaldehyde (Merck, catalog no. P6148) for 15 min at room temperature, permeabilized with 0.2% Triton X-100 (Merck, catalog no. T8787-250ML) for 30 min, washed three times with PBS, and incubated in 3% BSA for 1 h. Samples were then sequentially incubated with primary antibodies (E-Cadherin, Cell Signaling Technology, catalog no. 14472; N-Cadherin, Cell Signaling Technology, catalog no. 14215) overnight at 4°C, washed three times with PBS, and then fluorescent-conjugated with secondary antibodies for 50 min at room temperature. Nuclei were stained with DAPI (Merck, catalog no. 10236276001) for 15 min at room temperature. Slides were examined with an Axio Imager Upright Microscope (Carl Zeiss, Axio Imager M2).

### Cell Viability and Proliferation Assays

Mutated 22Rv1 cells treated with DHT or ethanol (2 × 10^3^ per well) were seeded in 96-well plates. Cell viability and proliferation were determined with XTT assays (Roche, 11465015001) at designed time points by measuring the absorbance at 450 nm, following the manufacturer’s instructions. Values were obtained from three replicate wells for each treatment and time point. The results were representative of three independent experiments. Significance was calculated by a two-tailed *t* test.

### Western Blots

Cell pellets were harvested and resuspended in lysis buffer (600 mM NaCl, 1% Triton X-100 in PBS, 1 × protease inhibitor). The protein extracts were separated by electrophoresis in a 12% polyacrylamide gel before transferring to PVDF membrane. Membranes were blocked with 5% nonfat milk in TBST for 1 h at room temperature. Incubation with primary antibodies was performed at 4°C overnight (E2F1, ABclonal, catalog no. A19579; MYC, ABclonal, catalog no. A17332; TERT, ABclonal, catalog no. A2979; V5, Invitrogen, catalog no. R960-25; E-Cadherin, Cell Signaling Technology, catalog no. 14472; N-Cadherin, Cell Signaling Technology, catalog no. 14215; GAPDH, ABclonal, catalog no. AC002). Membranes were washed three times with TBST every 10 min before secondary incubation with antibodies fused to horseradish peroxidase (HRP) (goat anti-mouse IgG, ABclonal, catalog no. AS003; goat anti-rabbit IgG, Thermo Fisher Scientific, catalog no. 31460) for 1 h at room temperature. After three final washes, Chemiluminescence signal was developed with SuperSignal™ West Pico Plus Sensitivity Substrate (Thermo Fisher Scientific, catalog no. 34094).

### Invasion Assay

Cells were detached by trypsinization and resuspended in serum free charcoal stripping media growth medium at 2.5 × 10^5^ cells/ml. Two hundred microliters of cell suspension with or without androgen treatment was transferred into 8-μm Transwell inserts (Corning Costar, catalog no. 3422) with 100 µl of Matrigel (diluted with serum free medium to 250µg/ml) coating (BD Biosciences, catalog no.356230). Charcoal stripping media growth medium (750 µl) with or without androgen was added in the lower chambers. After 48 h, 3.7% formaldehyde was used to fix the cells; after being permeabilized with methanol, cells were stained with Wright-Giemsa (WG16-500ml, Sigma). Cells on the upper surface of the membranes were removed and the cells at the bottom surface of the filters were quantified by counting the number of cells that penetrated the membrane in five microscopic fields (acquired at 20× magnification) per membrane. A two-tailed *t*-test was employed to perform statistical analysis from three replicate inserts.

### Chromatin Immunoprecipitation

The ChIP experiment was performed as previously described (16). Briefly, cells were crosslinked with 1% formaldehyde (Merck, catalog no. F8775) and the reaction was stopped with 125 mM glycine (Merck, catalog no. G8898-1KG). The nuclei were isolated from cells and then suspended in SDS lysis buffer. Nuclear extracts were sonicated to generate an average size of 400-bp chromatin fragment. In each reaction, 6 µg of indicated antibodies (E2F1, Invitrogen, catalog no. 32-1400; MYC, Invitrogen, catalog no. MA1-980) or control IgG (ABclonal, catalog no. AC011) was incubated with 70 µl of Dynabead protein G (10004D, Invitrogen) slurry in IP buffer for 10 h. Then, the supernatant was removed, and fragmented chromatin was diluted in 1.3 ml of IP buffer, which was added onto bead/antibody complexes. After 12 h of incubation, the complex was washed once with wash buffer I (20 mM Tris-HCl, pH 8.0, with 2 mM EDTA, 0.1% SDS, 1% Triton X-100, and 150 mM NaCl) and one time with buffer II (20 mM Tris-HCl, pH 8.0, with 2 mM EDTA, 0.1% SDS, 1% Triton X-100, and 500 mM NaCl), followed by two washes with buffer III (10 mM Tris-HCl, pH 8.0, with 1 mM EDTA, 250 mM LiCl, 1% Deoxycholate, and 1% NP-40) and buffer IV (10 mM Tris-HCl, pH 8.0, and 1 mM EDTA). DNA was purified with MinElute PCR Purification Kit (Qiagen, catalog no. 28006) and the target DNA fragments were analyzed by qPCR or direct Sanger sequencing of PCR fragments harboring these SNPs.

### Quantitative PCR

Each target fragment was amplified using SYBR Select Master Mix (Applied Biosystems, catalog no. 4472908). All target primers had three technical replicates, and the data were normalized to the control regions; then, the relative enrichment of the target antibodies at target DNA fragment was determined by comparison with the IgG control. Primer sequences used in this experiment can be found in **Supplementary Table 2**.

### Wound Healing Assays

Cells were seeded into 24-well plates and allowed to grow to near confluency. P200 pipette tip was used to scrape the cells and the same field was imaged after 72 h. To induce AR activity, the cells were treated with 100 nM DHT. The area of the wound in each well was analyzed using ImageJ. Results are representative of three independent experiments. Statistical significance was calculated by the two-tailed *t* test.

### Single-Nucleotide Mutation Using CRISPR/Cas9

The experiment was performed according to the previous protocol (17). Briefly, one pair of sgRNA (sgRNA1-top: CACCGCCAGGACCGCGCTTCCCACG, sgRNA1-bottom: AAACCGTGGGAAGCGCGGTCCTGGC) was designed (https://portals.broadinstitute.org/gpp/public/analysis-tools/sgrna-design) and inserted into pSpCas9 (BB)-2A-Puro (PX459) (Addgene plasmid ID: 48139). rs2853669 (T or C) centered DNA fragments were cloned into pGL3 basic vector to generate repair templates (**Supplementary Table 1**). Three hundred nanograms of indicated Cas9 plasmid [pSpCas9 (sgRNA) with sgRNA] and 300 ng of pGL3 vector with repair templates were co-transfected into 22Rv1 cells using Lipofectamine 2000. Medium was changed 24 h later. Puromycin (0.8 µg/ml) (Merck, catalog no. P9620) was added onto transfected cells after 48 h. After non-transfected cells were killed by puromycin, the remaining cells were sorted using flow cytometry to establish single-cell clones. The single cells were seeded in 96-well plates and checked for 9–14 days to rule out the non-single clone. Finally, the single clones were picked up for subculture and genotyping.

### Electrophoretic Mobility Shift Assays

The experiment was performed with LightShift^®^ Chemiluminescent EMSA kit (Thermo Fisher Scientific, catalog no. 20148), following the manufacturer’s instructions. Full length of *E2F1* or *MYC* was cloned into vector pcDNA3.1/V5-HisA (Invitrogen, catalog no. V81020). Briefly, double-stranded biotin-labeled consensus DNA was incubated with HEK293T cell nuclear extract with ectopically expressed E2F1 or MYC in a 1× binding buffer, 50 ng/µl poly(dI:dC)·poly(dI:dC), 2.5% glycerol, 0.05% NP-40, 5 mM MgCl_2_, and 10 mM EDTA, and 200-fold excess of unlabeled probes was used for competition assays. The protein complexes were resolved on 6% DNA retardation gels for 1 h at 100 V, transferred to Biodyne B Nylon Membranes (PALL, catalog no. 60208), cross-linked, and detected using streptavidin-HRP conjugate and a chemiluminescent substrate. The oligos used are listed in **Supplementary Table 3**.

### Lentiviral Constructs, Lentivirus Production, and Infection

The shRNA constructs targeting *E2F1* and *MYC* were ordered from Merck. The shRNA sequences used are listed in **Supplementary Table 4**. Second-generation lentiviral vectors were packaged using HEK293T cells. In detail, HEK239T cells were trypsinized and seeded into 3.5-cm plates; 24 h later, the normal medium was replaced with 2 ml of low-glucose DMEM (Invitrogen, catalog no. 21885025) containing 10% FBS and 0.1% penicillin and streptomycin. Cells were co-transfected with indicated shRNA construct (1.5 μg each), pMD2.G (envelope plasmid, 0.375 μg) (Addgene, catalog no. 12259), and psPAX2 (packaging plasmid, 1.125 μg) (Addgene, catalog no. 12260) plasmids using 8 µl of Lipofectamine 2000 (Invitrogen, catalog no.11668019). The medium was changed to fresh medium after 24 h, and the virus-containing medium was harvested every 24 h up to three times. Lentivirus was passed through a 0.45-μm filter unit and stored at −80°C. For viral transduction, LNCaP cells were seeded in six-well plates at a density of 60%–70%. Sixteen to 20 h later, cell culture medium was replaced with lentivirus-containing medium with final 8 μg/ml polybrene (Merck, catalog no. H9268). For lentivirus-mediated knockdown experiment, virus was removed and replaced by normal medium containing final 1 μg/ml puromycin (Merck, catalog no. P9620) after 24 h. When uninfected control cells were completely killed, the target cells were cultured in normal growth medium with 0.5 μg/ml puromycin.

### Motif Analysis

The effect of rs2853669 on transcription factor binding motifs was analyzed using R package at SNP v1.2.0 (affinity test for regulatory SNP detection) (18) in R (v.3.6.3). Binding affinity tests were performed for the motif matches between MYC and E2F1 with alleles of rs2853669 using the derived motif library ENCODE. R packages “seq Logo” (v. 1.52.0) (19) and “universal motif” (v. 1.4.10) (20) were applied to create and plot motif logos.

### Genotype Imputation

IMPUTE2 (v. 2.3.2)^64^ was used to perform the genotype imputation of rs2853669 from three cohorts, TCGA, Stockholm, and Cambridge, composed of 389, 94, and 119 prostate samples, respectively. QCtool (v.2.0.7) (20) was used to assess and perform quality control by setting parameters “-threshold 0.9, -snp-stats”. SNPs that failed to pass the quality checking were excluded from imputation. 1000 Genomes Phase 3 data were selected as reference panel (18). Parameters were set as default “–Ne = 2000 and –k hap = 500”. A SNP-centered 2-MB region was set for the imputation on chromosome 5. Genotypes in the Gen format were converted to dosage format for downstream analysis. The transcriptional profiling was assessed by Illumina Expression Bead Chip in Swedish and Cambridge human prostate tissue samples, while RNA-seq was used in TCGA samples. The Stockholm and TCGA cohorts were genotyped on Illumina Omni 2.5 and Affymetrix SNP array 6, respectively.

### RNA-Seq and Differential Expression Analysis

Mutated T/T cells and C/C cells were harvested, total RNA was extracted with Trizol Reagent (Roche, catalog no. 11667165001) and ethanol precipitation, and then samples were sent to BGI Group for sequencing. For the RNA sequencing of the T/T and C/C genotyped cells, single-end raw sequence reads were first pre-processed with FastQC (21) to assess the read quality. SOAPnuke (22) was employed to process reads for quality trimming and adapter removal with the following criteria: Reads with adaptors were removed, reads with more than 5% of Ns were filtered out, and low-quality reads with more than 20% of bases with quality score smaller than 10 were ruled out. Cleaned reads were further trimmed to 50 bp. A final FastQC run was performed to ensure the success of previous quality control steps. The processed reads were aligned against the human genome assembly hg38 using STAR version 2.7.2a (23) with default settings. HTSeq (htseq-count) was employed to quantitate aligned sequencing reads against gene annotation from Encode and with parameters “-s no, –i gene_name”. Differential expression analysis was performed from read count matrix using Bioconductor package DESeq2 (1.26.0) (24). Genes with low expressions (<2 cumulative read count across samples) were filtered out prior to differential expression analysis. A threshold of FDR < 0.05 was applied to generate the differentially expressed gene list. Data were normalized using variance Stabilizing Transformation (VST) method from DESeq2. A sample-to-sample distance matrix using hierarchical clustering with Euclidian distance metric from normalized total transcriptome of each sample was applied to examine expression correlations among biological replicates. Heatmap displaying differentially expressed genes between C/C and T/T was generated using the R package “pheatmap” (1.0.12). We have deposited the RNA-seq data to European Nucleotide Archive (ENA) with study accession number PRJEB47829.

### Gene Set Enrichment Analysis

We applied Gene Set Enrichment Analysis (GSEA) to interpret the biological mechanisms underlying the RNA-Sequencing of T/T and C/C genotyped cells from differential expression results obtained from DESeq2. The pre-ranked gene list was obtained by sorting the “stat” statistics from the differential gene list in a descending order. GSEAPreranked test (25) was applied to examine the enrichment of upregulated genes in C/C allele compared to T/T allele from the MSigDB database including Hallmark gene sets, WikiPathways gene sets, chemical and genetic perturbations gene sets, and Biocarta gene sets. Parameters were set as follows: Enrichment statistic = “weighted”, Max size (exclude larger sets) = 5,000, number of permutations = 1,000. All other parameters were kept as default. The GSEA enrichment plots were generated using R packages “clusterProfiler” (3.14.3) (26) and “enrichplot” (1.6.1) (27).

### Generation of the CC Genotype Signature

The CC genotype signature was developed from the differentially expressed genes upon C/C *vs.* T/T alleles from RNA-seq. A list of 311 genes was initially generated by applying cutoff with FDR ≤ 0.01 and *log_e_* ≥ 2 to the 5016-DE gene list. We defined this 311-gene list as CC genotype signature and acquired available gene expression data from cBioportal, which further resulted in a 205-gene signature. We then calculated the *Z* score value of this signature by summing up the normalized gene expression data and further examined its correlation with AR signaling, CCP, hypoxia, and EMT scores.

### Statistical Analysis

All statistical analyses were performed using RStudio (v. 1.2.5033) (28) with R (v. 3.6.3) (29). Data were obtained from the cBioPortal for Cancer Genomics (30, 31), Oncomine database (32), and GEO database (33, 34). Differential gene expression analyses were applied among normal prostate, tumor, and metastatic tissues from various independent cohorts. Statistical analyses were performed to study the correlation between gene expression levels and clinical features including lymph nodes. Statistical tests for patients with gene expression defined to two groups were calculated using Mann–Whitney *U* test, while Kruskal–Wallis *H* test was applied for cohorts with more than two groups. Kaplan–Meier survival analysis was used to assess the impact of gene expression levels on PCa prognosis and survival. Patients were stratified based on median expression of genes or genotype of rs2853669. For the association between rs2853669 genotype and the prognosis survival, we tested several scenarios considering the synergistic effects of gene expression levels and rs2853669 genotype. The survival analyses were performed and visualized as Kaplan–Meier plots by using the R package “Survival” (v. 3.2.3) (35, 36) and “Survminer” (v. 0.4.7) (37). Function “Surv” was first used to generate the survival models with “time-to-event” and “event status” as input from clinical cohorts. Median expression of genes was further followed to fit the models by function “survfit”. Statistical analyses for all Kaplan–Meier curves were calculated using log-rank test. Cox proportional hazards model was employed to assess the hazard ratio (HR) (38). To examine the association of expression of *TERT*, *E2F1*, and *MYC* with androgen signaling, we devised a representative AR signaling signature with a panel of 10 genes, including *SOX9*, *RAN*, *TNK2*, *EP300*, *PXN*, *NCOA2*, *AR*, *NRIP1*, *NCOR1*, and *NCOR2*. The *Z* score sum of these 10 genes was calculated, and patients were grouped by the median expression of the AR signaling signature. For correlation analysis of gene expressions, we tested the linear correlation among the expression levels of *TERT*, *MYC*, and *E2F1* in benign prostate and tumor issues in several independent cohorts from cBioPortal and Oncomine databases. Both Pearson and Spearman methods were applied to assess the co-expression correlations between gene expression levels. For microarray-based expression profiling, we selected gene probes with lowest *p-*values. Samples with missing genotype, expression, or patient survival data were excluded from analyses. *p-*value < 0.05 was considered to be statistically significant. Statistical tests and figures were generated in R (3.6.3)

## Results

### Discovering an Association of the *TERT* Promoter Variant rs2853669 With PCa Susceptibility

**Figure 1:**
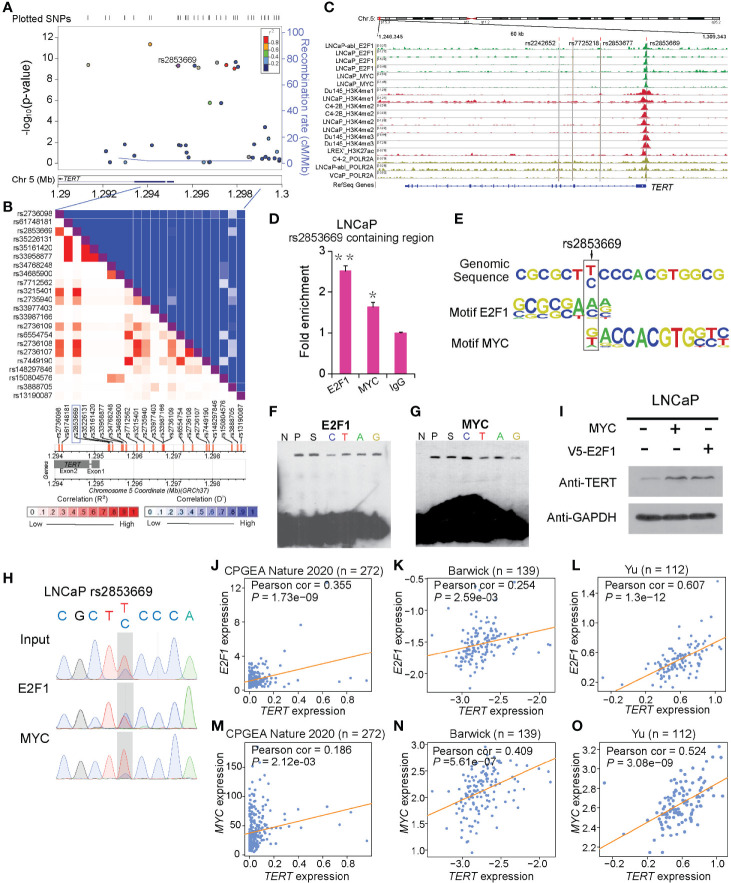
rs2853669 modulated E2F1 and MYC binding to the *TERT* promoter region. **(A)** Regional plot of the associated SNPs at chr5:1.29mbp−1.30mbp. The plot showed the –log_10_ *p*-values of association between the SNPs in this region and PCa. The intensity of red shading indicated the strength of LD (*r* ^2^) with the index SNP (rs2853669). **(B)** LD matrix plot indicated the LD pattern in the 50-kb region around the 5’-UTR region of *TERT* based on 1000 Genome CEU population. **(C)** ChIP-seq enrichment signals showing the bindings of E2F1 and MYC at the *TERT* promoter region. **(D)** ChIP-qPCR confirmation of the chromatin bindings of E2F1 and MYC at the rs2853669-containing region in LNCaP cells over background levels using a nonspecific IgG antibody. **(E)** rs2853669 resided within the DNA-binding motifs of E2F1 and MYC. E2F1 was predicted to preferentially bind to C allele of rs2853669, while MYC favored T allele of rs2853669. **(F, G)** The C allele of rs2853669 showed stronger binding affinity for E2F1 than the T allele in an electrophoresis mobility shift assay (EMSA) **(F)**. The T allele of rs2853669 showed stronger binding affinity for MYC than the C allele in an EMSA **(G)**. Lane N represented no protein extract for DNA to bind. The binding of the consensus sequence to E2F1 **(F)** or MYC **(G)** (lane P) was competed by a scrambled sequence (lane S) and by sequences containing the C allele (lane C) and T allele (lane T) of rs2853669 or permutations of an A base (lane A) or G base (lane G) at the same chromosomal location as rs2853669. **(H)** E2F1 or MYC preferred the binding to C allele or T allele at rs2853669 respectively confirmed by ChIP followed by Sanger sequencing in LNCaP cells. **(I)** Overexpression of MYC or E2F1 in LNCaP cells elevated the protein level of TERT by Western blot. **(J–O)** Scatterplots showing a positive expression correlation between *TERT* and *E2F1* **(J)** or *MYC* **(K)** in prostate tumor tissues. Error bars, s.e.m. *n* = 3 technical replicates. *p < 0.05, **p < 0.01. The *p-*values were assessed using two-tailed Student’s *t* tests.

To investigate whether *TERT* expression was affected by SNPs, we performed an association study and fine mapping analysis in several independent populations (see Methods) using the SNPs resided in *TERT* or far from *TERT* 5′ promoter region. We found several SNPs associated with PCa risk; however, among these SNPs, only one SNP named rs2853669 (located at 245 bp to ATG site of *TERT*) was residing in the functional region. Fine mapping showed strong linkage disequilibrium (LD) between rs2853669 and SNPs at 5’-UTR, but not the coding region of *TERT* (**Figures 1A, B**). Interestingly, our results indicated an opposite association of C or T allele of rs2853669 with PCa in different populations. The C allele of rs2853669 was significantly associated with PCa susceptibility (odds ratio, OR = 1.10 (95% CI 1.07–1.13), *p* = 4.76 × 10^-10^, **Table 1**) in Caucasians from UK Biobank (10,207 cases and 199,534 controls), and the T allele of rs2853669 was considered as a risk allele [OR = 1.29 (95% CI 1.01–1.64), *p* = 0.038, **Table 1**] in the Chinese population (1,100 cases and 1,410 controls). In addition, we observed the similar OR value of T allele at rs2853669 in another Chinese population cohort (39) even though the *p*-value is not significant [OR = 1.28 (95% CI 0.89–1.83), *p* = 0.18, **Table 1**]. Thus, the regulatory mechanism underlying rs2853669 needs to be deeply investigated to explain these contradictory results.

**Table 1:** The association between rs2853669 variants and PCa or disease aggressiveness.

Phenotypes of PCa	rs2853669 genotypes	UK Biobank-CEU (*n* = 209,741)		China (*n* = 2,510)		China (*n* = 2,425) (39)	
		OR (95% CI)	*p*-value	OR (95% CI)	*p*-value	OR (95% CI)	*p*-value
PCa	Additive	1.10 (1.07–1.13)	4.76E−10	1.10 (0.97–1.25)	0.12	1.14 (0.97–1.34)	0.10
	Dominant	1.12 (1.07–1.16)	1.02E−07	1.07 (0.89–1.27)	0.49	1.14 (0.97–1.34)	0.10
	Resessive	–	–	1.29 (1.01–1.64)	0.038	1.28 (0.89–1.83)	0.18

Reference allele: T. Alternative allele: C.

UKB-case/control: 10,207 cases vs. 199,534 controls.

Chinese: 1,100 cases vs. 1,410 controls.

Chinese: 1,417 cases vs. 1,008 controls (39).

### rs2853669 Altered Allele-Specific Chromatin Binding of E2F1 and MYC

To shed light on the mechanisms underlying rs2853669 in PCa, we first sought to find whether there might be oncogenic transcription factors binding at this SNP-containing region. We thus observed ChIP-seq enrichment signals of MYC and E2F1 at the rs2853669-containing region, and this observation was further verified in LNCaP cells (**Figures 1C, D**). We next performed SNPs and transcription factor DNA-binding motif matching analysis (40) and examined whether variation at rs2853669 directly modulates transcription factor binding. This analysis revealed that rs2853669 maps within the binding motifs of both E2F1 and MYC (**Figure 1E**). Interestingly, E2F1 had a higher preference for the C allele whereas MYC favored the T allele of rs2853669. To confirm this allele-specific DNA binding, we performed electrophoretic mobility shift assays (EMSAs) with HEK293T cell nuclear extract containing ectopically expressed E2F1 or MYC, respectively. Consistent with the motif analysis results, we observed the DNA binding preference of E2F1 to the C or A allele and MYC to the rs2853669 G or T allele (**Figures 1F, G**). In line with *in silico* motif analysis and *in vitro* EMSA results, our ChIP-qPCR followed by Sanger sequencing results further proved that MYC or E2F1 preferentially bound to different alleles of rs2853669 *in vivo* in LNCaP cells (**Figure 1H**). Altogether, we demonstrated that both MYC and E2F1 can bind at the *TERT* promoter region, and MYC prefers binding to T allele while E2F1 favors the C allele of rs2853669.

### *TERT* Was a Potential Target Gene of E2F1 and MYC

To assess whether MYC or E2F1 affects the expression of TERT, we performed both ectopic overexpression and short hairpin RNA mediated knockdown of MYC or E2F1 assays in PCa cells. The results showed that the protein level of TERT was upregulated upon overexpression of MYC or E2F1 and downregulated upon knockdown of MYC or E2F1 (**Figure 1I** and **Supplementary Figures S1A–D**). Furthermore, we observed significant positive correlations between the mRNA expression levels of *TERT* and *MYC* or *E2F1* respectively in multiple large cohorts of clinical prostate tissue samples (**Figures 1J–O** and **Supplementary Figures S1E–J**), suggesting that MYC and E2F1 regulate the expression of *TERT* in the clinical settings. Interestingly, our genome-wide co-expression analysis revealed that *TERT* is the target gene in both TGCA and CPGEA cohorts (**Supplementary Figures S1K, L**), whereas *TERT* is a top-ranking target gene positively correlated with *E2F1* in one cohort (41) but not with *MYC*, while in the other cohort (42), *TERT* and *MYC* were highly positively correlated, and no significant expression correlation was observed between *TERT* and *E2F1* (**Supplementary Figures S1M, N**). These results further indicated that MYC or E2F1 might regulate *TERT* expression through different regulatory mechanisms.

### The Role of E2F1 in Regulating MYC Disturbed by AR Signaling Pathway

**Figure 2:**
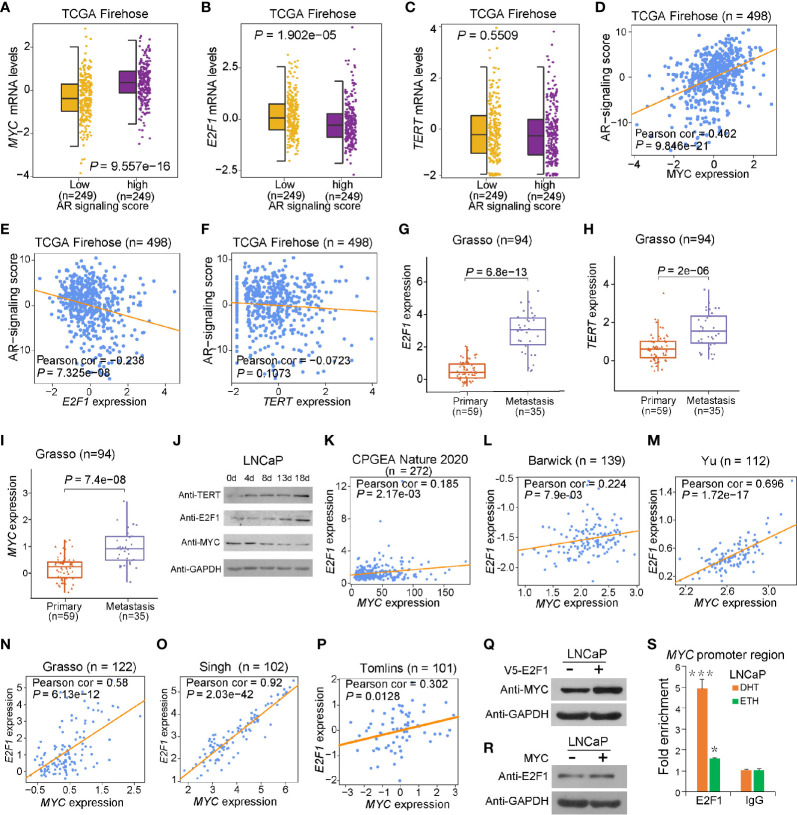
Effect of AR singling on *E2F1*, *MYC*, and *TERT* expression. **(A**, **D)** *MYC* was upregulated in the group with high AR signaling signature score (*SOX9*, *RAN*, *TNK2*, *EP300*, *PXN*, *NCOA2*, *AR*, *NRIP1*, *NCOR1*, and *NCOR2*). MYC was significantly positively correlated with AR signaling score. **(B**, **E)** *E2F1* was downregulated in AR signaling high group compared to AR signaling low group. *E2F1* was negatively correlated with AR signaling score. **(C, F)** No significant correlation was observed for *TERT* upon the AR signaling signature in the TCGA cohort. The *Z* score sum of the ten-gene AR signaling signature was stratified based on the median score. *p-*values were calculated using Mann–Whitney *U* test. **(G–I)** mRNA levels of *E2F1* **(G)**, *TERT* **(H)**, and *MYC* **(I)** were elevated in human benign, primary, and metastasis PCa in the Grasso cohort. *p-*values were calculated using Kruskal–Wallis test. **(J)** Western blot showing protein levels of TERT, E2F1, and MYC in LNCaP cells with hormone-deprived medium for the indicated days. GAPDH was used as a loading control. **(K–O)** Expression correlation of *MYC* with *E2F1* in human prostate tissues. Scatter plot showing the direct correlation between *MYC* with *E2F1* expression in the CPGEA cohort (*n* = 272) **(K)**, Barwick cohort (*n* = 139) **(L)**, Yu cohort (*n* = 112) **(M)**, Grasso cohort (*n* = 122) **(N)** Singh cohort (*n* = 102) **(O)**, and Tomlins (*n* = 101) **(P)**. **(Q, R)** Western blot showing the protein level of MYC in LNCaP cells overexpressing V5 tagged E2F1 **(P)** and E2F1 expression level in LNCaP cells overexpressing MYC plasmid **(Q)**. GAPDH was used as a loading control. **(S)** ChIP-qPCR results showed E2F1 chromatin binding at *MYC* promoter region in LNCaP cells. Error bars, s.e.m. n = 3 technical replicates. *P < 0.05, ***P < 0.001, The P values were assessed using two-tailed Student’s *t* tests.

Given that there are close regulatory associations between AR signaling and MYC or E2F1 in prostate tumors (43–45), we investigated the correlation between AR signaling intensity and the expression of *MYC*, *E2F1*, or *TERT*. We observed higher *MYC* and lower *E2F1* expression levels in the PCa patient group with higher AR signaling activity compared to that of lower AR signaling group (**Figures 2A, B, D, E** and **Supplementary Figures S2A–D**). No clear expression correlation between *TERT* and AR signaling was observed despite the fact that *TERT* was regulated by MYC and E2F1 (**Figures 2C, F** and **Supplementary Figures S2E, F**). Moreover, *E2F1* expressions were elevated in metastasis prostate tumors in several independent cohorts of PCa (**Figure 2G** and **Supplementary Figures S2G–I**). *TERT* expressions showed an elevated trend in metastasis prostate tumors in several independent cohorts of PCa even though the significance was not strong in Taylor and Yu datasets (**Figure 2H** and **Supplementary Figures S2J–L**). In contrast, the expressions of *MYC* in metastasis prostate tumors in different cohorts were inconsistent (**Figure 2I** and **Supplementary Figures S2M–O**). These inconsistent results might be attributable to the differences in AR signaling pathway in the metastasis prostate tumors. Therefore, the association of *MYC*, *E2F1*, and *TERT* with androgen-dependent PCa progression to androgen-independent stage needs to be deeply investigated.

Thus, we next cultured LNCaP cells with hormone-deprived medium for 18 days and analyzed the expression of MYC, E2F1, and TERT at five time points (**Figure 2J**). Notably, the MYC level gradually decreased during androgen deprivation, whereas TERT and E2F1 progressively increased during androgen deficiency for 18 days. Next, we aimed to examine the regulatory correlation between *MYC* and *E2F1*. Notably, a significant transcriptional expression correlation between *MYC* and *E2F1* was observed (**Figures 2K–P**). Our validation experiments showed that ectopic expression of E2F1 markedly elevated MYC protein expression; in contrast, ectopic expression of MYC showed no effect on E2F1 protein level (**Figures 2Q, R**). This was consistent with our observations that MYC protein level was significantly downregulated upon knockdown of E2F1, while knockdown of MYC had no impact on E2F1 protein level (**Supplementary Figures S1C, D**). In addition, it was reported that E2F1 physically interacts with AR (46), suggesting that the regulatory association between E2F1 and MYC might correlate with AR signaling pathway. We thus treated PCa cells with or without dihydrotestosterone (DHT) and performed ChIP-qPCR experiment. The results demonstrated that E2F1 has an apparent stronger binding at *MYC* promoter region in cells with DHT treatment than in control cells (**Figure 2S**), indicating an androgen signaling-dependent manner. Taken together, in androgen-responsive cells, E2F1 might cooperate with AR signaling pathway to promote TERT expression through upregulating the expression level of MYC when cells were under higher androgen stimulation. However, in an androgen deprivation environment, E2F1 could directly upregulate TERT expression without the involvement of MYC to assist androgen-dependent cells’ survival in adverse environments.

### AR Signaling Pathway Coordinated rs2853669-Mediated Regulation of *TERT* Expression Through E2F1 and MYC

**Figure 3:**
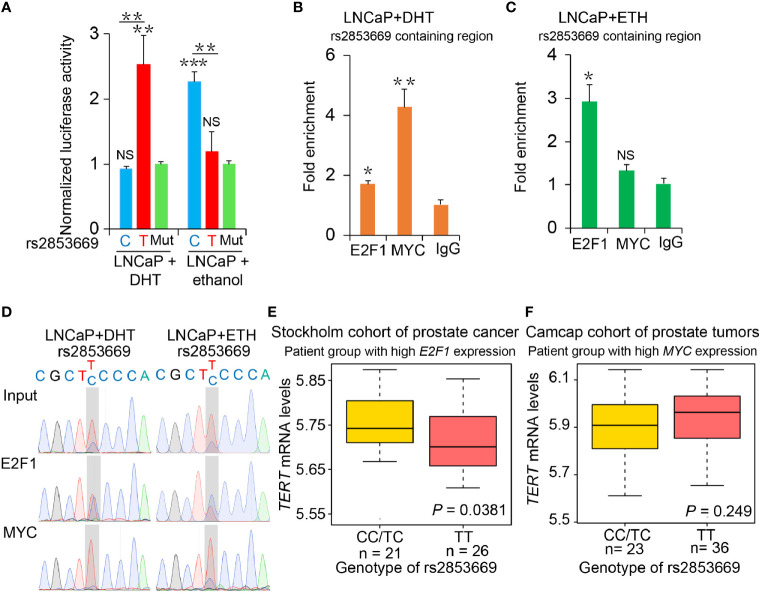
The binding preference of E2F1 and MYC at rs2853669 affected *TERT* expression. **(A)** Luciferase reporter assays showing increased promoter activity of the T allele at rs2853669 relative to the C allele in LNCaP cells after androgen treatment. The promoter activity of the T allele at rs2853669 relative to the C allele was diminished in LNCaP cells after removing androgen. Mut, deletion of MYC or E2F1-binding site with rs2853669. **(B, C)** ChIP-qPCR results showed MYC and E2F1 chromatin binding at rs2853669-containing region in LNCaP cells under androgen stimulation **(B)** or withdrawal **(C)**. **(D)** MYC or E2F1 favored binding to the T or C allele at rs2853669 with or without androgen treatment determined by ChIP followed by Sanger sequencing. **(E, F)** The association between rs2853669 genotype and *TERT* expression in prostate tumor samples. C allele of rs2853669 was significantly associated with elevated mRNA expression of *TERT* in *E2F1* high expression group **(E)**; Homozygous TT genotype of rs2853669 was correlated with higher expression of *TERT* in *MYC* high expression group **(F)**. Patients were pre-stratified based on median expression of *E2F1* or *MYC*. *p-*values were examined by a log-rank test. Error bars, s.e.m. *n* = 3 technical replicates. *p < 0.05, **p < 0.01, ***p < 0.001, Student’s *t* tests. NS, Non significance.

These findings together lead us to an important assumption whether the regulatory mechanisms underlying MYC and E2F1 at the rs2853669 region were coordinated with the status of androgen signaling. To test this hypothesis, we performed luciferase-based promoter assays and observed that androgen stimulus obviously increased the promoter activity of the region harboring T allele but decreased the promoter activity of the region with the C allele in LNCaP and 22Rv1 cells, respectively. Conversely, the region harboring the C allele was observed with an increased promoter activity compared to the region with T allele under androgen deprivation conditions (**Figure 3A** and **Supplementary Figure S3A**). We then tested the promoter activity in RWPE1 cells; T allele increased the promoter activity when undergoing DHT treatment, but both the plasmid with T and C allele had no obvious luciferase activity in normal prostate epithelial cells (**Supplementary Figure S4B**). We further performed ChIP followed by quantitative PCR and Sanger sequencing in androgen-sensitive PCa LNCaP cells. Androgen stimulation significantly promoted MYC binding at the fragment harboring rs2853669, whereas androgen deprivation obviously increased the enrichment of E2F1 at this region (**Figures 3B, C**). Different from cancer cells, we did not observe the enrichment of either E2F1 or MYC at this region in normal RWPE1 cells, whereas MYC showed obvious enrichment at this SNP containing region when the cells were treated with DHT (**Supplementary Figures S4C, D**). Moreover, MYC was preferentially recruited to T allele at rs2853669 under DHT treatment, whereas E2F1 showed stronger binding affinity with C allele than T allele after androgen removal (**Figure 3D**).

Consistently, we observed that C allele at rs2853669 is associated with higher *TERT* expression in PCa patients with higher expression levels of *E2F1*, and *TERT* indicated a trend to be upregulated in patient group carrying T allele at rs2853669 with higher *MYC* expression levels (**Figures 3E, F**). There was no correlation between *TERT* expression and those two alleles at rs2853669 in patient group with lower E2F1 or MYC expression levels (**Supplementary Figures S3E, F**). To further provide evidence to support our results, we overexpressed E2F1 or MYC in T/T and C/C mutated cells; ectopic overexpression of E2F1 in C/C cells could significantly enhance *TERT* expression level compared with that in T/T cells, while ectopic overexpression of MYC in T/T cells could significantly enhance *TERT* expression level compared with that in C/C cells (**Supplementary Figures S4F, G**). Taken together, these results suggested that T allele could promote *TERT* expression through increasing the binding of MYC to *TERT* promoter when PCa cells were androgen-stimulated. In contrast, *TERT* expression level was maintained through cooperating the C allele of rs2853669 with E2F1 under androgen-deficient environment in PCa cells.

### Direct Effects of rs2853669 on EMT and CRPC

**Figure 4:**
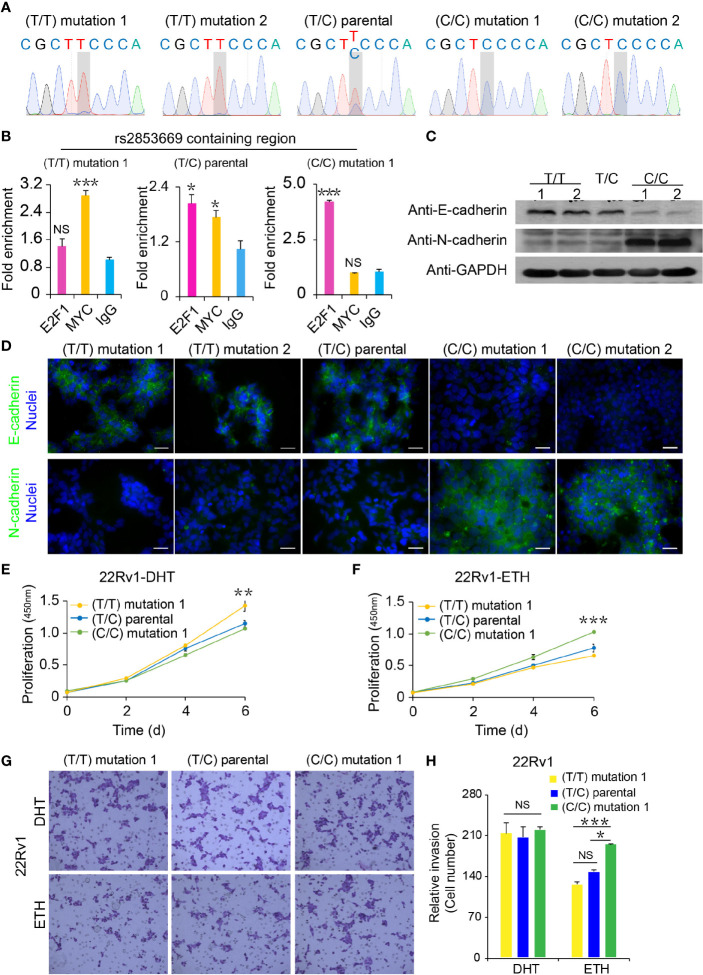
Functional analysis of CRISPR/Cas9-modified PCa cells with different rs2853669 genotype. **(A)** Sanger sequencing of CRISPR/Cas9-modified and parental 22Rv1 cells. **(B)** Chromatin enrichment of E2F1 and MYC at the rs2853669 site measured by ChIP-qPCR. **(C)** Western blot showing the protein levels of E-cadherin and N-cadherin in CRISPR/Cas9-modified and parental 22Rv1 cell lines. GAPDH was used as a loading control. **(D)** Representative microscopy analysis of E-cadherin (green; upper panels) and N-cadherin (green; lower panels) expression in CRISPR/Cas9-modified and parental 22Rv1 cell lines. Nuclei were counterstained with DAPI (blue). Scale bars, 20 μm. **(E, F)** Cell proliferation analysis of the TT genotype, TC genotype, and CC genotype at rs2853669 in 22Rv1 cells under androgen stimulation **(E)** or withdrawal **(F)**, mean ± SD of triplicate experiments. **(G, H)** Representative images of invasion **(G)** assays for cells under androgen stimulation or withdrawal. Scale bars, 100 µm. The number of cells in invasion **(H)** assays. ± s.e.m. from three biological replicates, *p < 0.05, **p < 0.01, ***p < 0.001, Student’s *t* test. NS, Non significance.

To further assess the phenotypic impacts of rs2853669 alteration, we first performed single-nucleotide mutation using CRISPR/Cas9-mediated genome editing approach and successfully converted the genotype of rs2853669 from T/C to T/T or C/C in the PCa cell line 22Rv1 (**Figure 4A**). We applied ChIP-qPCR to examine the occupancy status of MYC or E2F1 at the rs2853669-containing region in our mutated cells. We found that MYC chromatin occupancy at the rs2853669 locus was higher in the TT clones than in the TC and CC clones, while E2F1 chromatin occupancy at the rs2853669 locus was higher in the CC clone than the TC and TT clones (**Figure 4B**).

Although there were no obvious morphology differences among rs2853669 T/T, parental T/C, and C/C 22Rv1 cell lines (**Supplementary Figure S4A**), cadherin switching, a major hallmark of EMT (36), was observed among those clones. Downregulation of E-cadherin and upregulation of N-cadherin were confirmed by immunofluorescence and Western blot studies, indicating that C allele of rs2853669 played a pivotal role in EMT (**Figures 4C, D**). To explore whether androgen had influence on cellular proliferation and migration of the 22Rv1 cells with different genotypes of rs2853669, we treated cells with or without DHT and performed cell proliferation, wound healing, and invasion assays. T/T cells grow faster than T/C and C/C cells under DHT treatment (**Figure 4E**), whereas C/C cells grow faster than the other two genotyped cell clones after removing androgen (**Figure 4F**). Consistently, we found an obvious inhibition of wound closure and invasion ability in both T/T and T/C cells after removing androgen, while wound closure and invasion ability in C/C cells was not affected by androgen deficiency (**Figures 4G, H** and **Supplementary Figures S4B–E**). However, we did not observe obvious difference between the wound closure and invasion ability among T/T, T/C, and C/C cells when treated cells with DHT.

**Figure 5:**
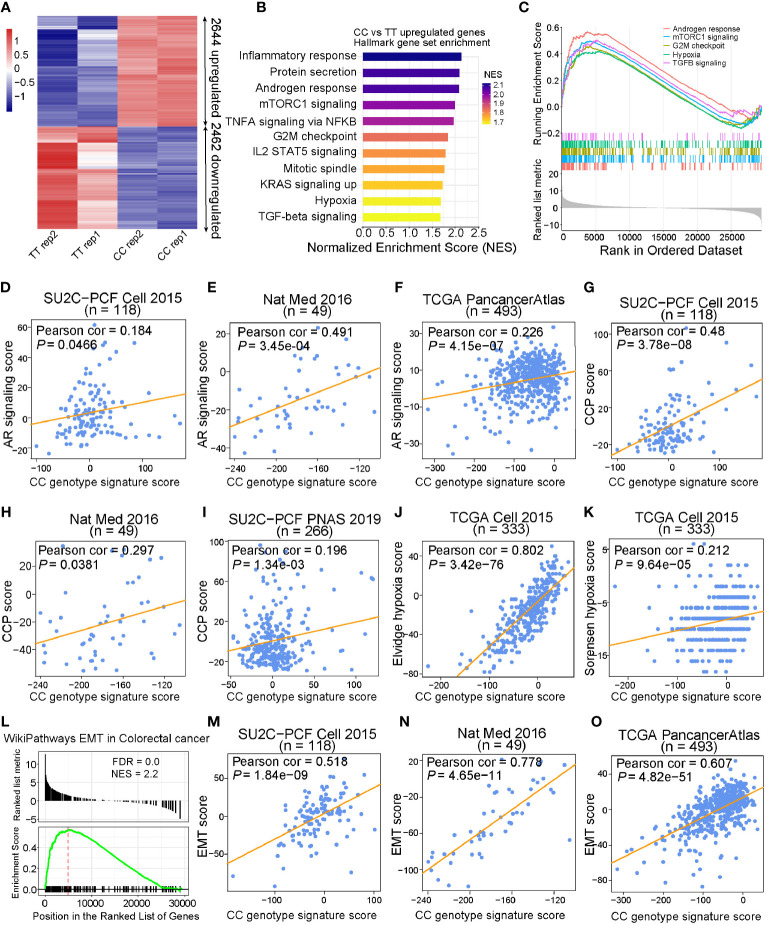
CC alleles upregulated cell cycle and EMT genes that are associated with cancer. **(A)** Heatmap of CC allele target genes measured by RNA-Seq (FDR < 0.05). **(B)** Hallmark gene sets enriched in genes upregulated by CC allele. Gene sets were ranked by normalized enrichment score with FDR < 0.01. **(C)** Gene Set Enrichment analysis (GSEA) of CC upregulated genes in Androgen response, mTORC1 signaling, G2M checkpoint, hypoxia, and TGFB signaling from Hallmark gene sets. **(D–F)** CC genotype gene signature score based on *z*-score sum of the 205 differentially expressed genes targeted by CC alleles revealed strong linear positive correlation with AR signaling score in SU2C-PCF, Nat Med, and TCGA cohorts. **(G–I)** Scatter plots displaying significant positive correlation between CC genotype signature score and cell cycle progression (CCP) score in SU2C-PCF, Nat Med, and SU2C-PCF PNAS cohorts. **(J, K)** Pearson correlation examination demonstrated that there was positive linear correlation between CC genotype signature score and hypoxia score in the TCGA cohort. **(L)** CC upregulated genes enriched in epithelial–mesenchymal transition (EMT) in colorectal cancer from WikiPathways. **(M–O)** CC genotype signature score showed strong linear positive correlation with EMT score in SU2C-PCF, Nat Med, and TCGA cohorts, respectively. Genes were ranked based on the statistics “stat” of DESeq2 result between CC and TT samples.

To further explore the underlying biological mechanisms that were affected by the different alleles at rs2853669, we next performed RNA sequencing (RNA-seq) analysis of T/T and C/C genotyped cells. Two biological replicates were performed in each group and high correlations between replicates were observed (**Supplementary Figure S5A**). The differential gene expression analysis identified 2,644 and 2,462 significantly upregulated and downregulated genes, respectively (FDR < 0.05; **Figure 5A**). Gene Set Enrichment Analysis (GSEA), performed in various gene sets from MSigDB, identified multiple pathways relevant to cell cycle and cancer development significantly enriched in upregulated genes of C/C cells compared to control cells with T/T alleles. Several cell growth-related genes in androgen response, hypoxia, Met, and EGF pathways were found upregulated in C/C cells (**Supplementary Table S5**, **Figures 5B, C**, and **Supplementary Figures S5B, C**). To explore the role of targeted genes affected by C/C alleles at rs2853669, we developed a CC genotype signature score and examined its relevance in various independent PCa cohorts. We found significant positive linear correlation between CC genotype signature and AR signaling (**Figures 5D–F** and **Supplementary Figures S5D–F**), cell cycle progression (CCP) (**Figures 5G–I**), and hypoxia scores (**Figures 5J, K** and **Supplementary Figures S5G, H**), which was in line with the results showing that C/C cells possessed more proliferative capability (**Figure 4F**). Moreover, in concordance with the above experimental results (**Figures 4G, H**), we found that genes involved in EMT, metastasis, and TGF-beta pathways were also upregulated in C/C cells (**Figure 5L** and **Supplementary Figures S5I, J**). The correlation analysis also revealed remarkable positive association between CC genotype signature score and EMT score in multiple PCa cohorts (**Figures 5M–O** and **Supplementary Figures S5K–M**), further indicating a higher invasiveness capacity of C/C cells compared to T/T cells. Collectively, our data validated that CC alleles maintain cell survival in hormone deficiency environment and play a crucial role in EMT progression.

### Synergistic Co-Overexpression of *TERT*, *E2F1*, and *MYC* Was Associated With Escalated Tumor Malignancy

**Figure 6:**
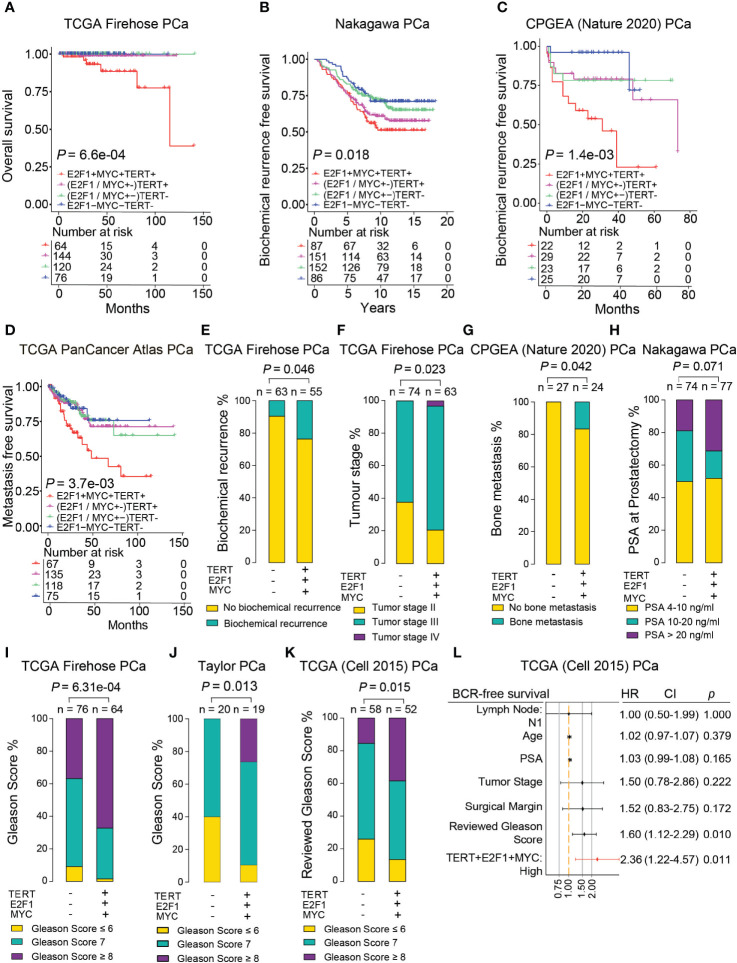
Synergistic co-overexpression of *TERT*, *E2F1*, and *MYC* correlated with escalated tumor malignancy. **(A–D)** Synergistic triple high expression of *TERT*, *E2F1*, and *MYC* was associated with poorer overall survival **(A)**, higher risks for biochemical relapse **(B, C)**, and poorer metastatic-free survival **(D)** in PCa patients. **(E–K)** Proportion of PCa patients with biochemical relapse **(E)**, advanced tumor stage **(F)**, bone metastasis **(G)**, higher PSA levels **(H)**, and higher Gleason Score **(I–K)** was significantly higher in the group with triple high (+) expression of *TERT*, *E2F1*, and *MYC*. **(L)** Multivariate Cox regression analysis examined the synergistic effect of co-overexpression of *TERT*, *E2F1*, and *MYC* together with other clinicopathological features on the risk of BCR-free survival in PCa patients in TCGA cohort.

Although MYC and E2F1 are two well-studied oncogenic transcription factors that are frequently dysregulated in PCa cells (47–49), whether the two genes together with TERT have synergistic effects on tumor severity are still unclear. To examine the synergistic effect of these three tumor oncogenic genes on patient survival time, we performed Kaplan–Meier analysis in several independent PCa cohorts. We found that patients with simultaneous triple high expression of *TERT*, *E2F1*, and *MYC* were significantly associated with shorter overall survival, elevated risks of biochemical relapse, and metastasis. In contrast, the PCa patient group with simultaneous triple low expression appeared with better prognosis (**Figures 6A–D** and **Supplementary Figures S6A, B**). Notably, this observation was not found in normal prostates of the CPGEA cohort (**Supplementary Figure S6C**). To further investigate the synergistic effects of the three genes in clinical settings, we examined its correlation with several clinical features including biochemical recurrence, tumor stage, metastasis, PSA, and patient neoplasm status in PCa cases. The results revealed that percentages of patients with biochemical relapse were significantly higher in groups with triple high expression of the three oncogenes (**Figure 6E** and **Supplementary Figure S6D**). The percentages of tumor stage III and IV were 62.2% and 79.4% in the low or high expression groups, respectively (*p* = 0.023) (**Figure 6F**). Patients carrying tumors were also substantially more in the triple high group (**Supplementary Figures S6E, F**). Proportions of patients with metastasis or PSA higher than 20 ng/ml were notably elevated in triple high group (**Figures 6G, H** and **Supplementary Figure S6G**). Moreover, the percentage of patients with advanced Gleason score was significantly higher in the triple high group in multiple cohorts (**Figures 6I–K**). These findings suggested that synergistic triple high expression of *E2F1*, *MYC*, and *TERT* were greatly associated with PCa severity and poorer prognosis. We next performed the multivariate Cox regression model to investigate the incorporated gene signature of TERT, E2F1, and MYC together with clinical prognostic factors influencing survival in PCa patients. The results revealed that the performance of the risk of the triple overexpression of the gene signature had the highest hazard ratio (**Figure 6L**), indicating a superior prognostic value in PCa compared to other clinicopathological features. Taken together, these findings indicated that the synergistic co-overexpression of TERT, E2F1, and MYC in PCa is strongly associated with poor prognosis, tumor progression, metastasis, and patient survival.

### Allele-Specific Impact of rs2853669 on PCa Survival

**Figure 7:**
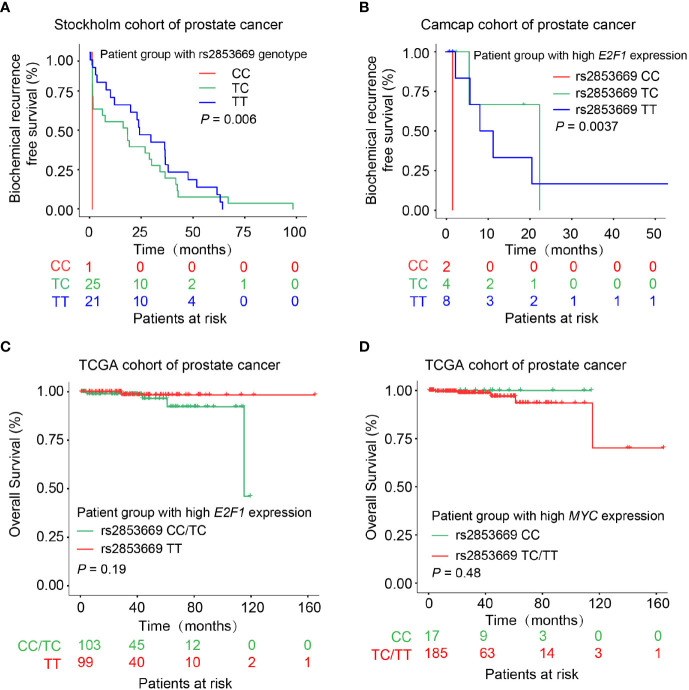
Effect of rs2583669 genotype on PCa patient prognosis. **(A, B)** Kaplan–Meier plots demonstrating the biochemical recurrence-free survival of PCa patients grouped by the genotype of rs2853669. Patients carrying C allele of rs2853669 correlated with increased risk for biochemical recurrence in the Stockholm cohort **(A)**. Patient group with high *E2F1* expression and carrying rs2853669 CC genotype underwent a higher biochemical recurrence risk **(B)**. **(C, D)** PCa patient group carrying rs2853669 C allele with higher *E2F1* expression tumors or T allele with higher *MYC* expression levels indicated an increased risk of overall survival. *p-*values were examined by a log-rank test.

We next asked whether the genotype of rs2853669 impacts PCa prognosis and thus examined the correlation of the CC genotype at rs2853669 with clinical features in PCa patients. This analysis showed that patients carrying genotype CC or TC at rs2853669 have a shorter time for biochemical relapse than patients with the TT genotype in the Stockholm cohort (**Figure 7A**). We also observed a clear trend in the CPGEA Nature 2020 dataset showing C allele associating with poor prognosis (**Supplementary Figure S7A**). Considering that E2F1 and MYC are essential factors driving the severity of various tumors (50, 51), we thus explored whether the correlation of rs2853669 genotype and clinical variables was affected by *MYC* or *E2F1* expression status. We observed that the CC genotype at rs2853669 was correlated with increased risk of biochemical relapse and overall survival rate in the PCa patient group with higher *E2F1* expression (**Figures 7B, C**). We additionally tested the association between the SNP and patient survival with the consideration of E2F1 expression levels in Stockholm and CPGEA Nature 2020 datasets shown in **Supplementary Figures S7B, C**. The results from both datasets showed that C allele is associated with poor prognosis. We observed a similar trend between the TT genotype at rs2853669, and the overall rate was observed in the patients with high expression of *MYC* (**Figure 7D**). These results further proved that E2F1 and MYC drive the progression of PCa through different alleles at rs2853669.

## Discussion

Over the past 15 years, GWASs have successfully identified many pleiotropic loci associated with PCa severity, despite the fact that a certain number of association results are inconsistent across different study cohorts. In this work, we also observed the opposite associations between rs2853669 and PCa in different populations. The C allele of rs2853669 was significantly associated with PCa severity observed in Caucasians from UK Biobank whereas T allele was found as a PCa risk allele in Chinese population.

We mechanistically determined that MYC and E2F1 regulate TERT expression in PCa *via* a regulatory element that is disrupted by the T allele or C allele at rs2853669, respectively. The switch with T or C allele playing roles in regulating *TERT* expression was determined by AR signaling though other unknown factors may not be ruled out. These findings suggested that either T or C allele at rs2853669 can be the risk allele when cells are under certain molecular conditions. Disease severity, hormone levels, and biological contexts among the clinical samples might cause different observations regarding the association between rs2853669 and PCa risk. Importantly, while upregulation of MYC by E2F1 was reported in other cancer types (52, 53), our findings deeply uncovered a critical role for E2F1 in regulating MYC expression in PCa providing a new perspective for exploring the crosstalk between E2F1 and MYC that function as two regulators in G1/S transition and tumor cell growth. Moreover, our study uncovered the critical role of C allele at rs2853669 in CRPC progression and EMT, dispensing new clues to unveil the molecular mechanisms underlying PCa development and progression. Both T and C alleles were essential for cell growth at the primary stage in PCa. However, C allele at rs2853669 showed higher risk association than that of T allele, implying that it plays a vital role in maintaining cell growth and enhancing invasion ability in hormone-free environment by recruiting E2F1. Future studies are required to discover whether there might be other factors affecting the regulatory mechanism of MYC and E2F1 at this SNP considering the biological context of individuals are unique and complicated. The androgen response genes were upregulated in C/C cells, thereby explaining why C/C cells had hormone castration resistant capacity in view of the fact that AR plays an important role in CRPC. In addition to androgen response genes, many growth and metastasis-associated genes were also upregulated in C/C cells, implying that the fragment harboring rs2853669 might be an enhancer element for other genes; alternatively, this SNP could change the three-dimensional structure of genome and may warrant further investigation.

Here, we proposed a novel model for studying germline variants in PCa; this model might be popular in other sex hormone relevant cancer types, such as breast cancer. For example, the roles of rs2853669 in breast cancer are still inclusive; one study reported an increased breast cancer risk in patients carrying the CC genotype (54), whereas other studies found that the CC genotype is not related to breast cancer risk (55). Our findings might provide more clues for unveiling the regulatory mechanisms underlying rs2853669 in breast cancer, and these inclusive results may be due to altered estrogen receptor signaling pathway, thereby facilitating us to fully understand the impact of the genetic variants on human diseases.

We reported here for the first time that the hormone level is one of the important factors in regulating the function of the variants. These results also proposed that there could be complicated crosstalk between one SNP and certain important signaling pathways in PCa. Complex factors such as ethnic groups, biological characters, genetic factors, and living surroundings between individuals brought more difficulties to explain the real biological functions of these loci. In future studies, detailed classification of tumor samples is essential because there might be more risk SNPs functioning like rs2853669 undiscovered. Moreover, more efficient genome editing tools need to be explored to convert the genotypes of more SNPs at one time to speed up the research of the synergies between two or more SNPs at the cellular level. Furthermore, the biological differences between individuals should also be considered when we implicate association studies to improve the detection, prognosis, and risk evaluation in PCa.

In summary, we interpreted the functional mechanisms of a 5p15 locus SNP rs2853669 in regulating *TERT* expression and PCa development, which may provide potential clues for improving PCa risk prediction and prognosis.

## Data Availability Statement

The original contributions presented in the study are included in the article/**Supplementary Material**. Further inquiries can be directed to the corresponding authors.

## Author Contributions

PG, RN, and G-HW supervised the project. XD, JH, QX, BX, PZ, and HL performed the experiments with the help from QH. RN performed cohort studies. TY provided advice on bioinformatics. QZ performed bioinformatics analysis. PG, RN, G-HW, XD, and QZ designed the studies and wrote the manuscript. All authors contributed to the article and approved the submitted version.

## Funding

This work was funded by the National Natural Science Foundation of China (81972417 and 82073082), Jane and Aatos Erkko Foundation, Sigrid Juselius Foundation, Natural Science Foundation of Shaanxi Province (2020JM-292 and 2020JQ-430), Fudan University recruit funds, the “1000 Young Scholars” Program of Shaanxi Province, Fundamental Research Funds for the Central Universities (GK201902002 and GK201903061), and College Students’ Innovative Entrepreneurial Training Plan Program (S202010718091).

## Conflict of Interest

The authors declare that the research was conducted in the absence of any commercial or financial relationships that could be construed as a potential conflict of interest.

## Publisher’s Note

All claims expressed in this article are solely those of the authors and do not necessarily represent those of their affiliated organizations, or those of the publisher, the editors and the reviewers. Any product that may be evaluated in this article, or claim that may be made by its manufacturer, is not guaranteed or endorsed by the publisher.
