# Supplementary material for: Large Multicohort Study Reveals a Prostate Cancer Susceptibility Allele at 5p15 Regulating TERT via Androgen Signaling-Orchestrated Chromatin Binding of E2F1 and MYC
Source: Front Oncol. 2021 Nov 10;11:754206. doi: 10.3389/fonc.2021.754206 (PMC8631195; doi:10.3389/fonc.2021.754206)
Supplement: Supplementary file 1 [file DataSheet_1.docx]

**Large multicohort study reveals a prostate cancer susceptibility allele at 5p15 regulating *TERT* via androgen signaling-orchestrated chromatin binding of E2F1 and MYC**


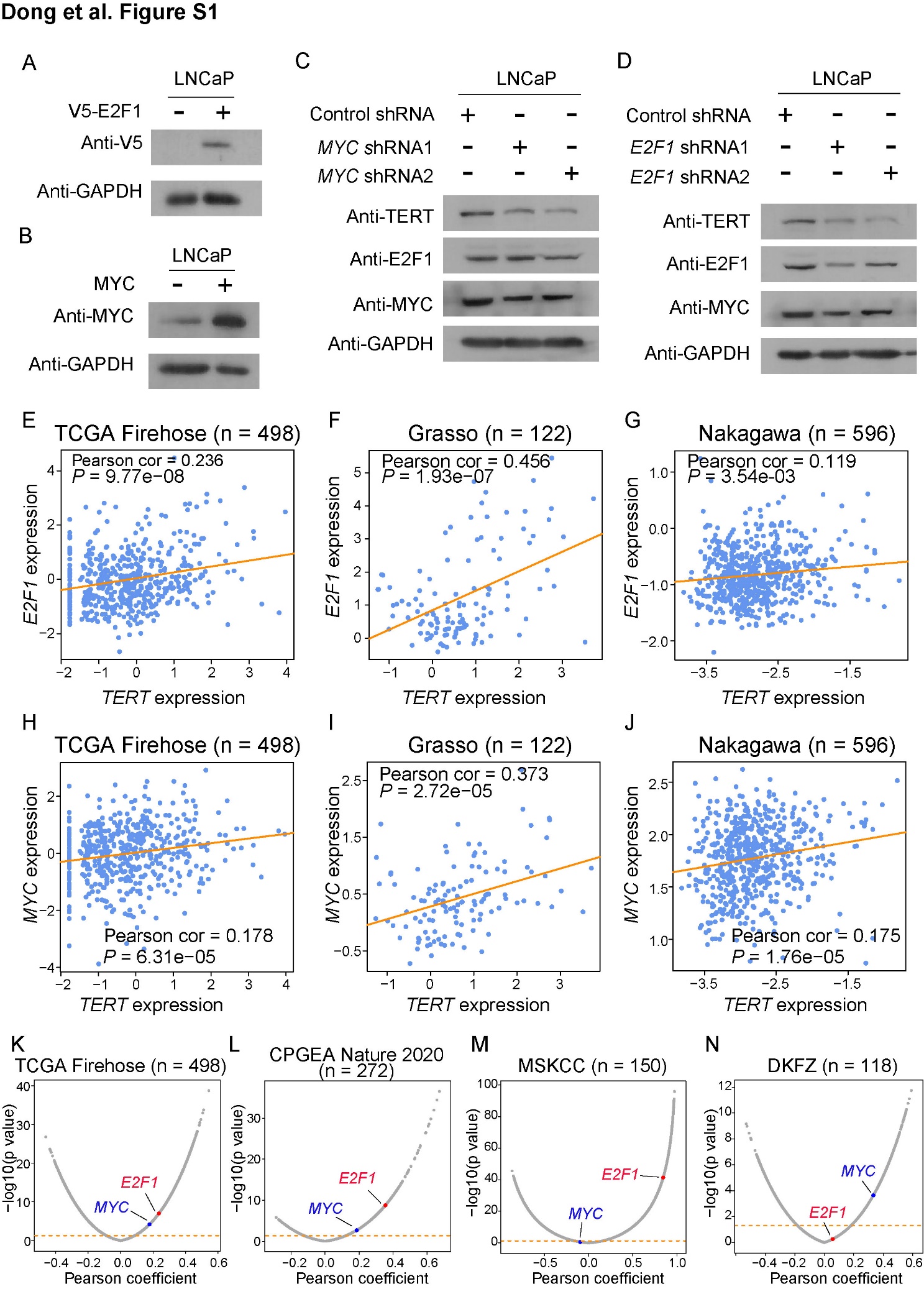


**Fig. S1 *TERT* was the target gene of MYC and E2F1.**

**A-B**, Immunoblotting of V5 tagged E2F1 (a) and MYC (b) overexpressed LNCaP cells with antibodies against V5 tag and MYC. GAPDH was performed as control. **C**, Knockdown of MYC using shRNAs resulted in significantly decreased expression level of TERT in LNCaP cells. GAPDH was used as a loading control. **D**, Depletion of E2F1 by shRNAs diminished the protein levels of TERT and MYC in LNCaP cells. GAPDH was performed as control. **E-J**, Expression correlation analysis displayed *TERT* significantly positively correlated with *E2F1* or *MYC* in TCGA, Grasso and Nakagawa prostate cohorts. **K-N**, A genome-wide co-expression analysis displaying the correlations between *TERT* and *E2F1* (red) or *MYC* (blue) in four independent cohorts, TCGA (K), CPGEA (L), MSKCC (M) and DKFZ (N). X-axis demonstrates Pearson coefficiency while Y-axis represents –log10 (*P* value).


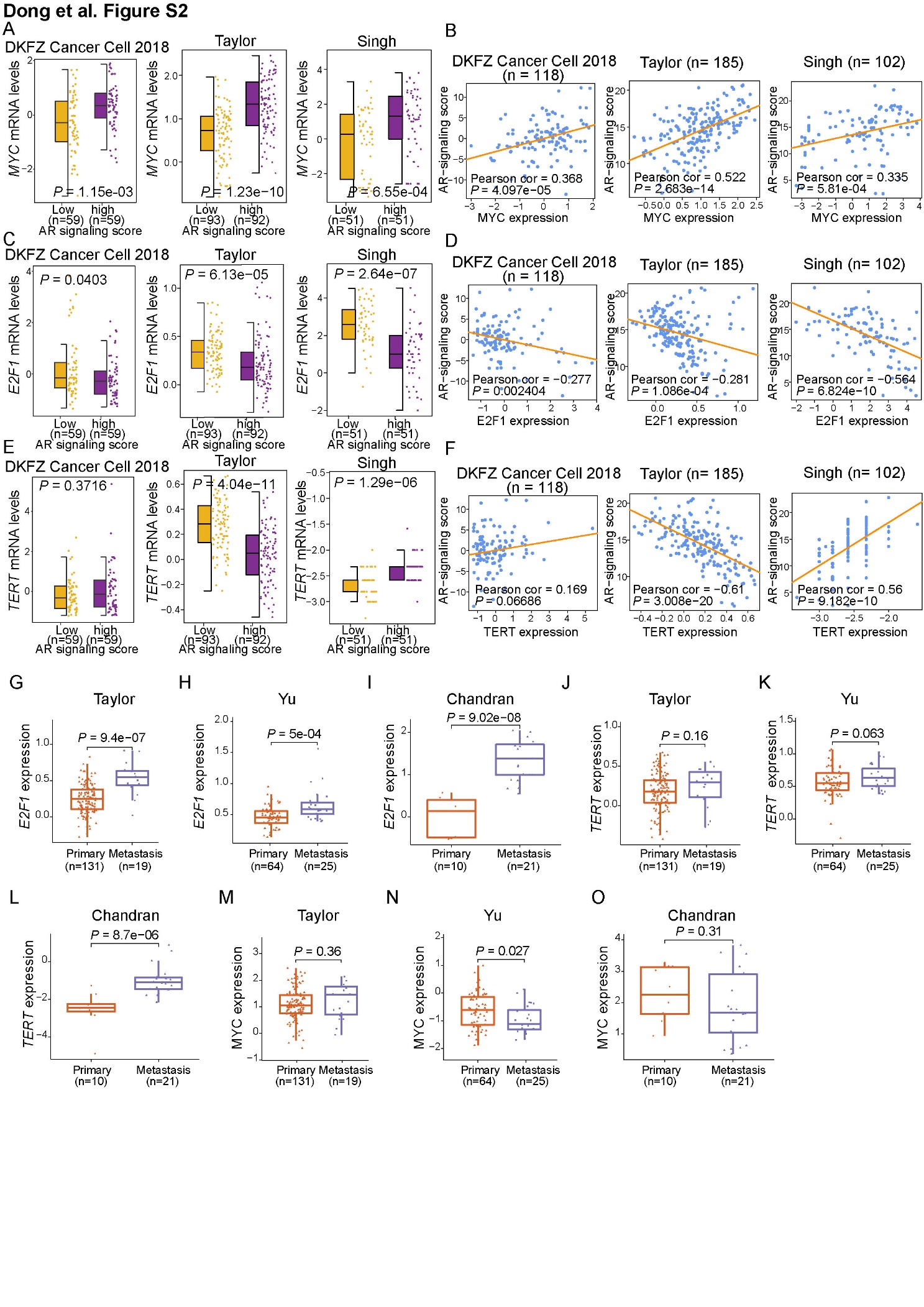


**Fig. S2 *E2F1* and *TERT* were highly expressed in advanced PCa samples. A-B**, *MYC* was upregulated in AR signaling high group compared to AR signaling low group. *P* values were calculated using Mann–Whitney U test. **C-D**, *E2F1* was downregulated in AR signaling high group compared to AR signaling low group. **E-F**, Inconsistent correlation was observed between TERT and the AR signaling signature. *P* values were calculated using Mann–Whitney U test. **G-I**, Elevated mRNA levels of *E2F1* in human benign, primary and metastasis PCa in Taylor(G), Yu(H) and Chandran(I) cohorts. **J-L**, Elevated trend of mRNA levels of *TERT* in human benign, primary and metastasis PCa in Taylor(J), Yu(K) and Chandan(L) cohort. **M-O**, MYC expression level was upregulated in metastasis PCa in Taylor (M) while downregulated in Yu(N) and Chandan(O) cohorts. *P* values were calculated using Kruskal-Wallis test for comparisons among three groups and Mann-Whiteney U test for two groups.


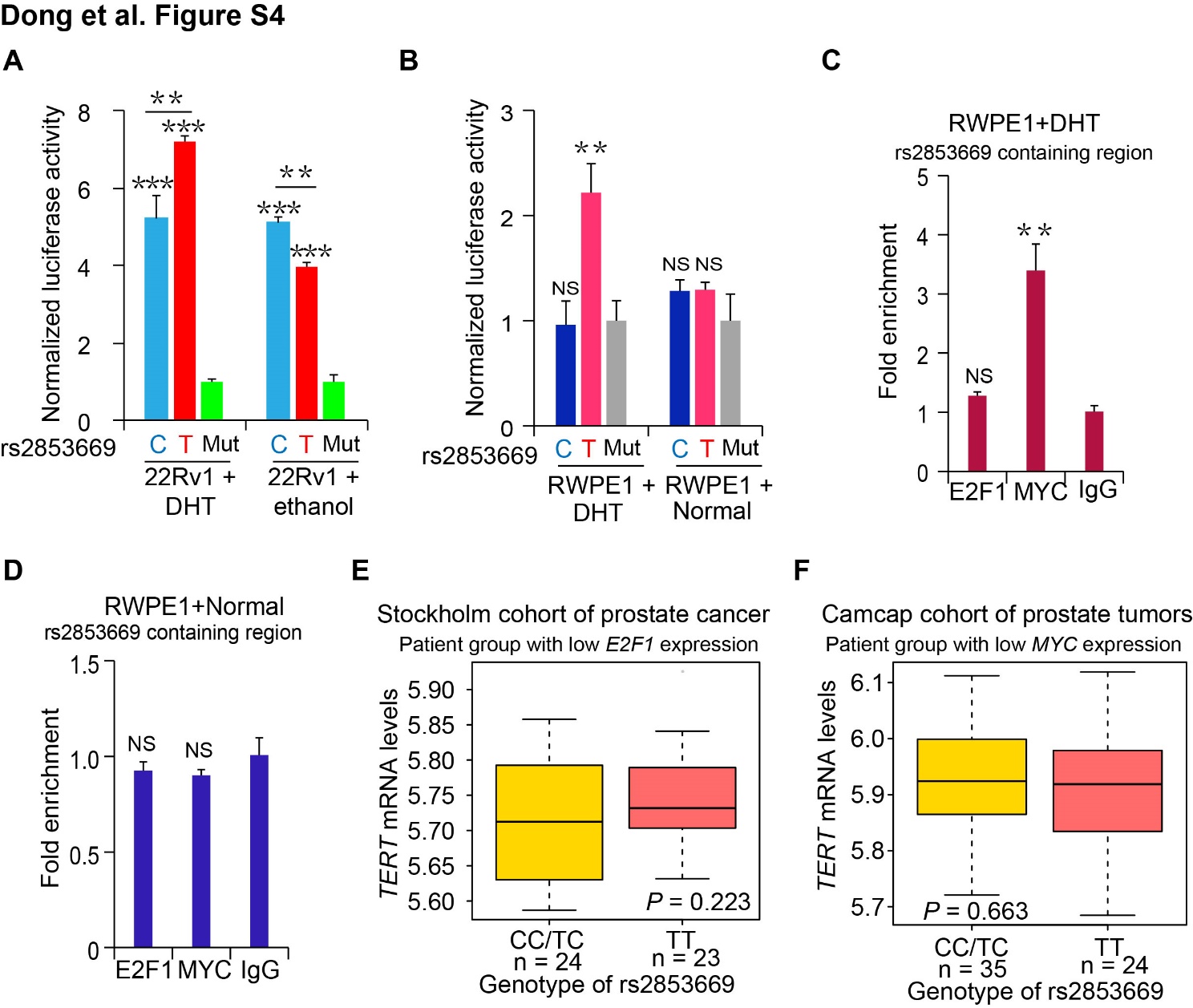


**Fig. S3 The effect of the binding preference of E2F1 and MYC at rs2853669 on *TERT* expression. A-B**, Luciferase reporter assays showing increased promoter activity of the T allele at rs2853669 relative to the C allele in 22Rv1(A) or RWPE1(**B**) cells after androgen treatment. The promoter activity of the T allele at rs2853669 relative to the C allele was diminished in 22Rv1 cells after removing androgen. Mut, deletion of MYC or E2F1-binding site with rs2853669. **C-D,** ChIP-qPCR results showed MYC and E2F1 chromatin binding at rs2853669-containing region in RWPE1 cells under androgen stimulation (**C**) or normal condition (**D**). Error bars, s.e.m. n = 3 technical replicates. ***p* < 0.01, ****p* < 0.001, Student’s *t* tests. **E**, C allele of rs2853669 was not associated with elevated mRNA expression of *TERT* in *E2F1* low expression group. **F**, Homozygous TT genotype of rs2853669 was not correlated with higher expression of *TERT* in *MYC* low expression group.


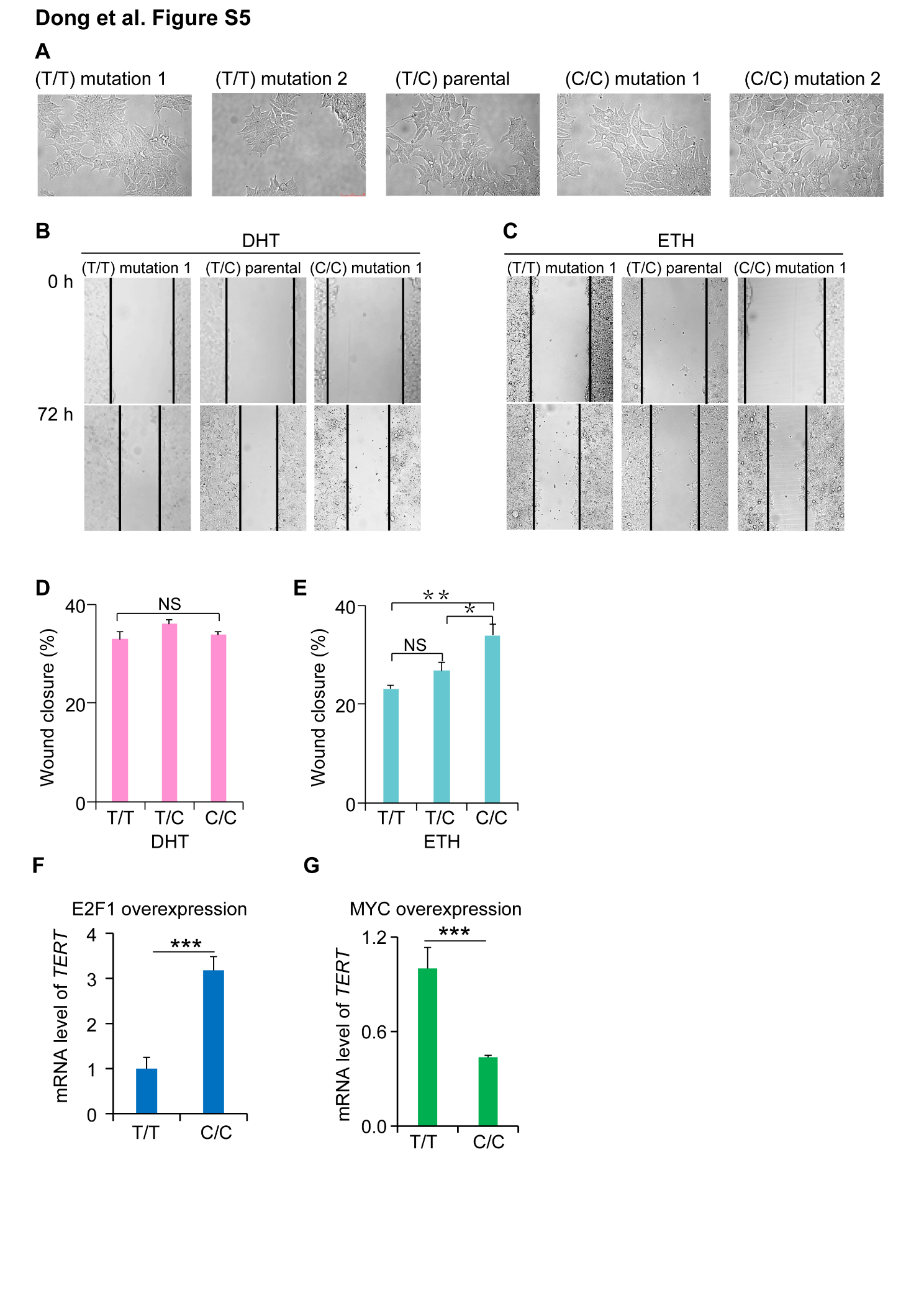


**Fig. S4** **CRISPR/Cas9 modified PCa cells had no genotype differences, but migration ability was varied.**  **A**, The phenotype of each 22Rv1 cell line was analyzed by microscopy under culture for two days. **B-C**, Representative images of scratch-wound assay for migration ability of 22Rv1 cells with different genotypes of rs2853669. **D-E**, Percentage fraction of original wound closure was determined from three independent wound healing experiments. **F-G**, Mutated cells (C/C or T/T cells) were transfected with MYC or E2F1 overexpression vector respectively. *TERT* mRNA level was detected by real time PCR. Statistical significance was assessed using two-tailed *t* test. *p*-values < 0.01 are signified by (**)


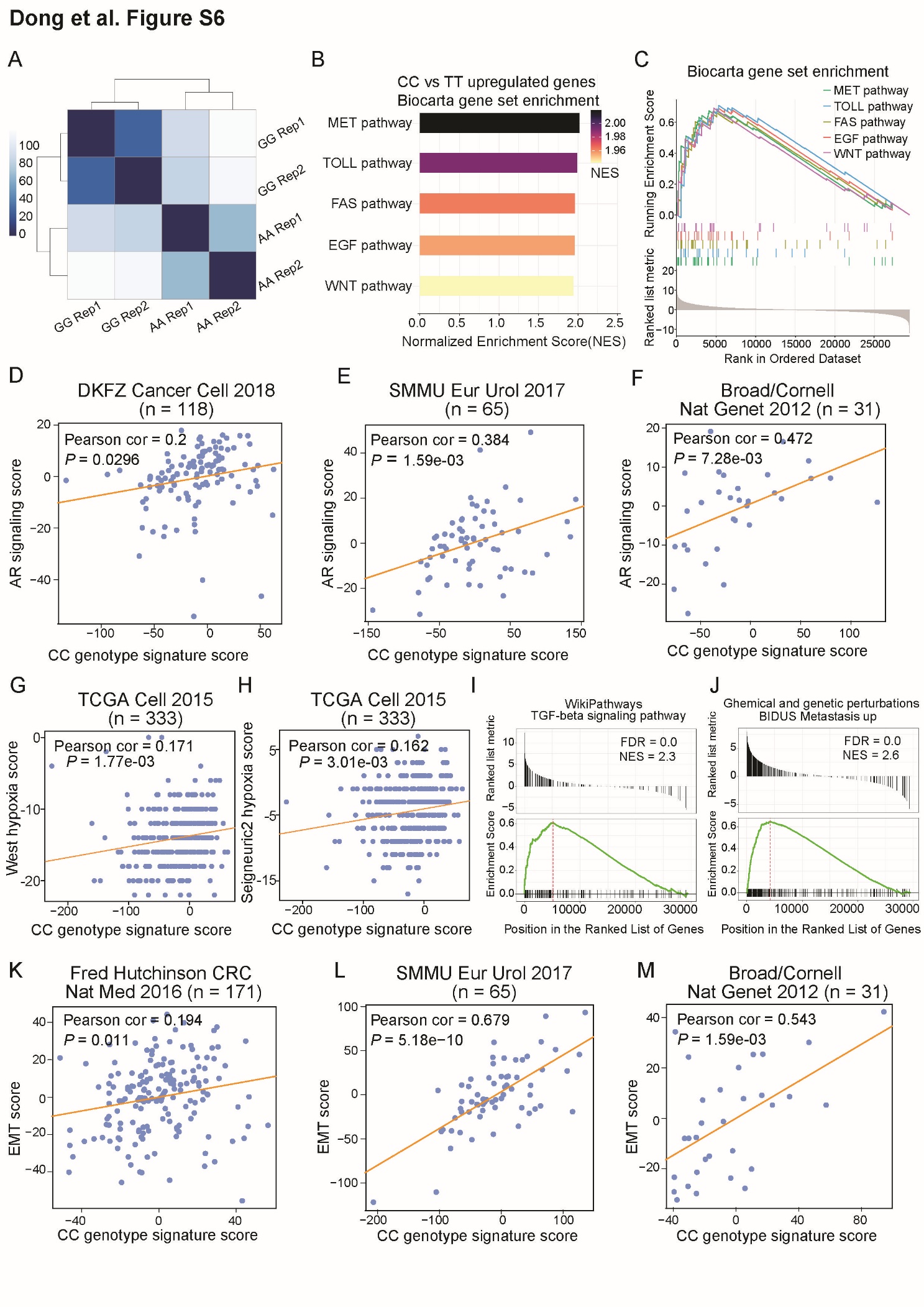


**Fig. S5 Quality-control metrics and pathway enrichment results from different MSigDB collections.**

**A**, Heatmap of sample-to-sample distance matrix using hierarchical clustering with Euclidian distance metric of normalized total transcriptome of each sample. The heatmap was built on normalized gene read counts using variance stabilizing transformation (VST) from DESeq2. **B**, Biocarta gene sets enriched in genes were upregulated by CC allele. Gene sets were ranked by normalized enrichment score with FDR < 0.01. **C**, Gene Set Enrichment analysis of CC upregulated genes in MET, TOLL, FAS, EGF and WNT signaling pathways from Biocarta gene sets. **D-F**, Scatter plots showing strong positive linear correlation between CC genotype signature score with AR signaling score. **G-H**, Pearson correlation revealed positive correlation between CC genotype signature score and hypoxia scores in different independent cohorts. **I-J**, CC upregulated genes enriched in TGF-beta signaling pathway from Wikipathways and BIDUS Metastasis up pathway enriched in Chemical and genetic perturbations. **K-M**, Scatter plots demonstrating significant positive correlation between CC genotype signature score and EMT scores in multiple independent PCa cohorts. Genes were ranked based on the statistics “stat” of DESeq2 result between CC and TT samples.


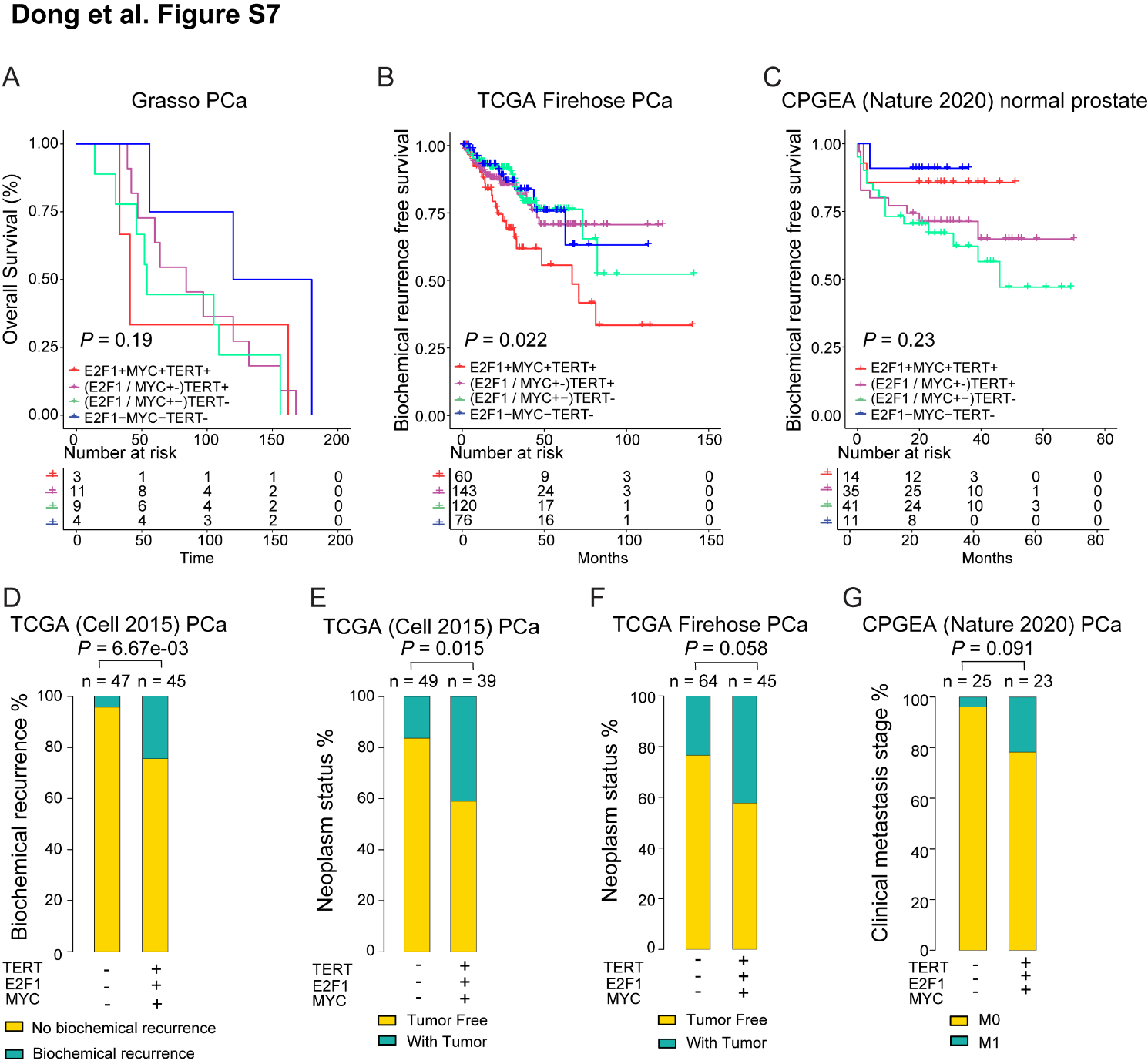


**Fig. S6 Clinical relevance of co-overexpression of E2F1, MYC, and TERT in normal prostate and tumours.**

**A**, Patients with triple high expression of *TERT*, *E2F1*, and *MYC* showed a trend for poor overall survival in Grasso cohort. **B**, Synergistic triple high expression of *TERT*, *E2F1*, and *MYC* was associated with poorer biochemical relapse in the TCGA cohort. **C**, No significant association was observed between biochemical relapse free survival and synergistic triple high expression of *E2F1* *MYC*, and *TERT* in normal prostates in the CPGEA cohort. **D-G**, Proportion of PCa patients with biochemical relapse (D), neoplasm (E-F) and metastasis was dramatically higher ingroup with triple high (+) expression of *TERT*, *E2F1*, and *MYC*.


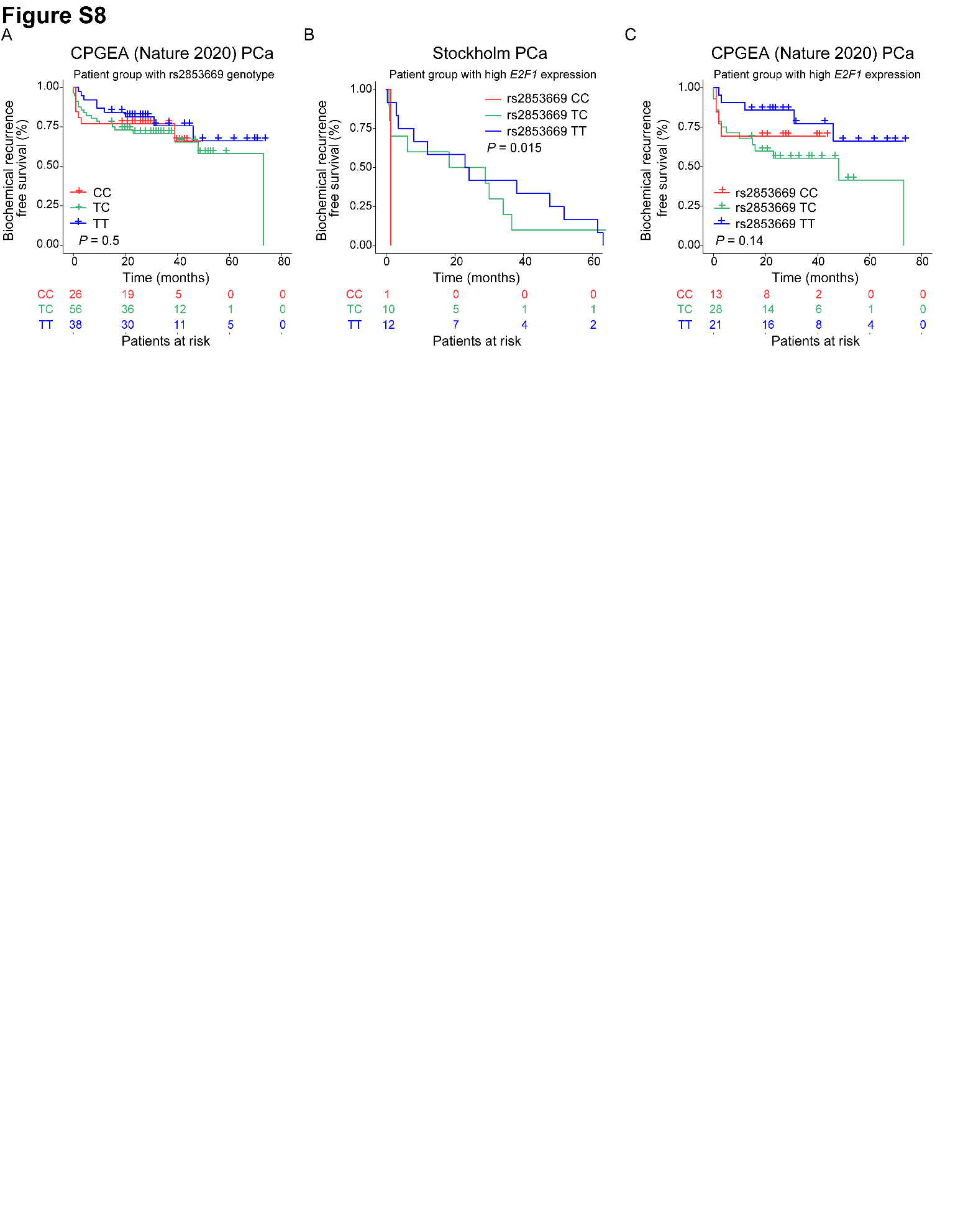


**Fig. S7 Effect of rs2853669 genotype on PCa patient prognosis.**

**A**, Kaplan-Meier plots demonstrating the biochemical recurrence-free survival of PCa patients grouped by the genotype of rs2853669. **B-C,** PCa patient group carrying rs2853669 C allele with higher E2F1 expression tumours indicated an increased risk of biochemical recurrence. *P* values were examined by a log-rank test.

**Table S1: Primers for cloning of cDNA and regulatory enhancer elements**

| **No.** | **Primer** | **Sequence 5' to 3'** | **Target** | **Vector** |
| --- | --- | --- | --- | --- |
| 1 | E2F1-F | CCCAAGCTTATGGCCTTGGCCGGGGCCCCT | E2F1 | pcDNA3.1 |
|  | E2F1-R | TGCTCTAGAGAAATCCAGGGGGGTGAGGTC |  |  |
| 2 | MYC-F | CGCGGATCCCTGGATTTTTTTCGGGTA | MYC | PLVET |
|  | MYC-R | CCGGAATTCTTACGCACAAGAGTTCC |  |  |
| 3 | Rs2853669-889F | GAAGATCTACGCACACCAGGCACT | rs2853669 | pGL3 Basic |
|  | Rs2853669-889R | CCCAAGCTTCTGTGTCAAGGAGCCCAAG |  |  |
| 4 | Rs2853669-530F | GAAGATCTCCTCCACATCATGGCCC | rs2853669 | pGL3 Basic |
|  | Rs2853669-530R | CCCAAGCTTGCACCTCGCGGTAGT |  |  |

**Table S2: Primers for quantitative real time PCR and sanger sequencing**

| **No.** | **Primer name** | **Sequence 5'-3'** |
| --- | --- | --- |
| 1 | Rs2853669-102F | CTGGAAGGTGAAGGGGCAG |
|  | Rs2853669-102R | GGGCTCCCAGTGGATTCG |
| 2 | MYCpro-94F | CGGGAAAAAGAACGGAGGGA |
|  | MYCpro-94R | CTGCCTCTCGCTGGAATTACT |
| 3 | ChIPNeg135-F | TGCCTCAGATTTGGAGTGCT |
|  | ChIPNeg135-R | GAGAAGCCTCTGAGGAGGGA |
| 4 | Rs2853669 Geno-235-F | CTGCCTGAAACTCGCGCC |
|  | Rs2853669 Geno-235-R | CTCCCAGTGGATTCGCGG |
| 5 | Actin-RT-F | AGAAAATCTGGCACCACACC |
|  | Actin-RT-R | AGAGGCGTACAGGGATAGCA |
| 6 | TERT-RT-F | CTCCTGCGTTTGGTGGATGA |
|  | TERT-RT-R | GGGCATAGCTGAGGAAGGTTT |

**Table S3: Oligos for Electrophoretic Mobility Shift Assays**

| **No.** | **Oligo Name** | **5´-> 3´Sequence** |
| --- | --- | --- |
| 1 | Bio-E2F1consG-F | CCGCCACGTGGGGAGCGCGGTCCT |
| 2 | E2F1scrambled1-R | CCGCACTTAATATTATATCGTCCT |
|  | E2F1scrambled1-R | AGGACGATATAATATTAAGTGCGG |
| 3 | E2F1snpT-F | CCGCCACGTGGGAAGCGCGGTCCT |
|  | E2F1snpT-R | AGGACCGCGCTTCCCACGTGGCGG |
| 4 | E2F2snpC-F | CCGCCACGTGGGGAGCGCGGTCCT |
|  | E2F2snpC-R | AGGACCGCGCTCCCCACGTGGCGG |
| 5 | E2F2snpA-F | CCGCCACGTGGGTAGCGCGGTCCT |
|  | E2F2snpA-R | AGGACCGCGCTACCCACGTGGCGG |
| 6 | E2F2snpG-F | CCGCCACGTGGGCAGCGCGGTCCT |
|  | E2F2snpG-R | AGGACCGCGCTGCCCACGTGGCGG |

**Table S4: shRNAs used in the project**

| **Name** | **Catalog** | **Manufacturer** |
| --- | --- | --- |
| Control-shRNA2 | SHC002 | Functional Genomics Unit (University of Helsinki) |
| MYC-shRNA1 | TRCN0000039640 | Merck |
| MYC-shRNA2 | TRCN0000174055 | Merck |
| E2F1-shRNA1 | TRCN0000039660 | Merck |
| E2F1-shRNA2 | TRCN0000010327 | Merck |

**Table S5:** **Hallmark gene sets enriched in genes upregulated by CC alleles**

| **Rank** | **Hallmark gene set enrichment** | **Size** | **ES** | **NES** | **NOM p-val** | **FDR q-val** | **FWER p-val** |
| --- | --- | --- | --- | --- | --- | --- | --- |
| 1 | [INFLAMMATORY RESPONSE](http://www.gsea-msigdb.org/gsea/msigdb/cards/HALLMARK_INFLAMMATORY_RESPONSE) | 150 | 0.55 | 2.13 | 0.000 | 0.000 | 0.000 |
| 2 | [PROTEIN SECRETION](http://www.gsea-msigdb.org/gsea/msigdb/cards/HALLMARK_PROTEIN_SECRETION) | 95 | 0.57 | 2.08 | 0.000 | 0.000 | 0.000 |
| 3 | [ANDROGEN RESPONSE](http://www.gsea-msigdb.org/gsea/msigdb/cards/HALLMARK_ANDROGEN_RESPONSE) | 100 | 0.57 | 2.08 | 0.000 | 0.000 | 0.000 |
| 4 | [MTORC1 SIGNALING](http://www.gsea-msigdb.org/gsea/msigdb/cards/HALLMARK_MTORC1_SIGNALING) | 198 | 0.50 | 1.99 | 0.000 | 0.000 | 0.000 |
| 5 | [TNFA SIGNALING VIA NFKB](http://www.gsea-msigdb.org/gsea/msigdb/cards/HALLMARK_TNFA_SIGNALING_VIA_NFKB) | 173 | 0.50 | 1.96 | 0.000 | 0.000 | 0.000 |
| 6 | [UV RESPONSE DN](http://www.gsea-msigdb.org/gsea/msigdb/cards/HALLMARK_UV_RESPONSE_DN) | 140 | 0.49 | 1.91 | 0.000 | 0.000 | 0.001 |
| 7 | [G2M CHECKPOINT](http://www.gsea-msigdb.org/gsea/msigdb/cards/HALLMARK_G2M_CHECKPOINT) | 200 | 0.46 | 1.84 | 0.000 | 0.001 | 0.005 |
| 8 | [IL2 STAT5 SIGNALING](http://www.gsea-msigdb.org/gsea/msigdb/cards/HALLMARK_IL2_STAT5_SIGNALING) | 171 | 0.45 | 1.79 | 0.000 | 0.003 | 0.014 |
| 9 | [ESTROGEN RESPONSE EARLY](http://www.gsea-msigdb.org/gsea/msigdb/cards/HALLMARK_ESTROGEN_RESPONSE_EARLY) | 191 | 0.44 | 1.78 | 0.000 | 0.002 | 0.015 |
| 10 | [MITOTIC SPINDLE](http://www.gsea-msigdb.org/gsea/msigdb/cards/HALLMARK_MITOTIC_SPINDLE) | 199 | 0.44 | 1.76 | 0.000 | 0.002 | 0.015 |
| 11 | [HEME METABOLISM](http://www.gsea-msigdb.org/gsea/msigdb/cards/HALLMARK_HEME_METABOLISM) | 185 | 0.44 | 1.75 | 0.000 | 0.002 | 0.016 |
| 12 | [KRAS SIGNALING UP](http://www.gsea-msigdb.org/gsea/msigdb/cards/HALLMARK_KRAS_SIGNALING_UP) | 151 | 0.44 | 1.72 | 0.000 | 0.002 | 0.020 |
| 13 | [INTERFERON ALPHA RESPONSE](http://www.gsea-msigdb.org/gsea/msigdb/cards/HALLMARK_INTERFERON_ALPHA_RESPONSE) | 89 | 0.47 | 1.72 | 0.000 | 0.002 | 0.021 |
| 14 | [UNFOLDED PROTEIN RESPONSE](http://www.gsea-msigdb.org/gsea/msigdb/cards/HALLMARK_UNFOLDED_PROTEIN_RESPONSE) | 111 | 0.46 | 1.71 | 0.000 | 0.003 | 0.024 |
| 15 | [APOPTOSIS](http://www.gsea-msigdb.org/gsea/msigdb/cards/HALLMARK_APOPTOSIS) | 151 | 0.44 | 1.70 | 0.000 | 0.002 | 0.025 |
| 16 | [COMPLEMENT](http://www.gsea-msigdb.org/gsea/msigdb/cards/HALLMARK_COMPLEMENT) | 160 | 0.43 | 1.68 | 0.000 | 0.003 | 0.036 |
| 17 | [HYPOXIA](http://www.gsea-msigdb.org/gsea/msigdb/cards/HALLMARK_HYPOXIA) | 188 | 0.42 | 1.68 | 0.000 | 0.003 | 0.036 |
| 18 | [TGF BETA SIGNALING](http://www.gsea-msigdb.org/gsea/msigdb/cards/HALLMARK_TGF_BETA_SIGNALING) | 53 | 0.50 | 1.67 | 0.004 | 0.004 | 0.042 |
| 19 | [APICAL JUNCTION](http://www.gsea-msigdb.org/gsea/msigdb/cards/HALLMARK_APICAL_JUNCTION) | 183 | 0.42 | 1.67 | 0.000 | 0.003 | 0.043 |
| 20 | [WNT BETA CATENIN SIGNALING](http://www.gsea-msigdb.org/gsea/msigdb/cards/HALLMARK_WNT_BETA_CATENIN_SIGNALING) | 38 | 0.53 | 1.66 | 0.011 | 0.003 | 0.046 |
| 21 | HEDGEHOG SIGNALING | 33 | 0.53 | 1.59 | 0.013 | 0.007 | 0.088 |
| 22 | EPITHELIAL MESENCHYMAL TRANSITION | 176 | 0.40 | 1.59 | 0.000 | 0.006 | 0.090 |
| 23 | CHOLESTEROL HOMEOSTASIS | 71 | 0.46 | 1.59 | 0.002 | 0.006 | 0.091 |
| 24 | ANGIOGENESIS | 29 | 0.54 | 1.59 | 0.010 | 0.006 | 0.092 |
| 25 | IL6 JAK STAT3 SIGNALING | 65 | 0.46 | 1.57 | 0.011 | 0.007 | 0.111 |
| 26 | APICAL SURFACE | 39 | 0.50 | 1.55 | 0.026 | 0.009 | 0.144 |
| 27 | E2F TARGETS | 200 | 0.38 | 1.53 | 0.002 | 0.010 | 0.161 |
| 28 | INTERFERON GAMMA RESPONSE | 171 | 0.39 | 1.52 | 0.002 | 0.010 | 0.176 |
| 29 | KRAS SIGNALING DN | 155 | 0.39 | 1.52 | 0.002 | 0.010 | 0.185 |
| 30 | GLYCOLYSIS | 192 | 0.37 | 1.47 | 0.002 | 0.015 | 0.262 |
| 31 | MYC TARGETS V1 | 200 | 0.36 | 1.44 | 0.005 | 0.020 | 0.342 |
| 32 | NOTCH SIGNALING | 32 | 0.48 | 1.44 | 0.056 | 0.021 | 0.370 |
| 33 | P53 PATHWAY | 192 | 0.35 | 1.43 | 0.005 | 0.022 | 0.391 |
| 34 | UV RESPONSE UP | 151 | 0.35 | 1.36 | 0.027 | 0.040 | 0.604 |
| 35 | XENOBIOTIC METABOLISM | 170 | 0.34 | 1.36 | 0.022 | 0.039 | 0.606 |
| 36 | COAGULATION | 108 | 0.36 | 1.32 | 0.050 | 0.054 | 0.737 |
| 37 | PANCREAS BETA CELLS | 30 | 0.44 | 1.30 | 0.109 | 0.059 | 0.773 |
| 38 | PI3K AKT MTOR SIGNALING | 96 | 0.35 | 1.29 | 0.060 | 0.065 | 0.811 |
| 39 | PEROXISOME | 102 | 0.33 | 1.21 | 0.126 | 0.121 | 0.954 |
| 40 | BILE ACID METABOLISM | 102 | 0.32 | 1.17 | 0.161 | 0.155 | 0.982 |
| 41 | FATTY ACID METABOLISM | 143 | 0.29 | 1.10 | 0.225 | 0.251 | 0.998 |
| 42 | ALLOGRAFT REJECTION | 139 | 0.28 | 1.06 | 0.314 | 0.312 | 1.000 |
